# Multidisease testing for HIV and TB using the GeneXpert platform: A feasibility study in rural Zimbabwe

**DOI:** 10.1371/journal.pone.0193577

**Published:** 2018-03-02

**Authors:** Zibusiso Ndlovu, Emmanuel Fajardo, Elton Mbofana, Tatenda Maparo, Daniela Garone, Carol Metcalf, Helen Bygrave, Kekeletso Kao, Sekesai Zinyowera

**Affiliations:** 1 Medecins Sans Frontières, Southern Africa Medical Unit, Cape Town, South Africa; 2 Medecins Sans Frontières, Access Campaign, Geneva, Switzerland; 3 Medecins Sans Frontières, Harare, Zimbabwe; 4 Foundation for Innovative New Diagnostics, Geneva, Switzerland; 5 National Microbiology Reference Laboratory, Ministry of Health and Child Care, Harare, Zimbabwe; The Ohio State University, UNITED STATES

## Abstract

**Background:**

HIV Viral Load and Early Infant Diagnosis technologies in many high burden settings are restricted to centralized laboratory testing, leading to long result turnaround times and patient attrition. GeneXpert (Cepheid, CA, USA) is a polyvalent near point-of-care platform and is widely implemented for Xpert MTB/RIF diagnosis. This study sought to evaluate the operational feasibility of integrated HIV VL, EID and MTB/RIF testing in new GeneXpert platforms.

**Methods:**

Whole blood samples were collected from consenting patients due for routine HIV VL testing and DBS samples from infants due for EID testing, at three rural health facilities in Zimbabwe. Sputum samples were collected from all individuals suspected of TB. GeneXpert testing was reserved for all EID, all TB suspects and priority HIV VL at each site. Blood samples were further sent to centralized laboratories for confirmatory testing. GeneXpert polyvalent testing results and patient outcomes, including infrastructural and logistical requirements are reported. The study was conducted over a 10-month period.

**Results:**

The fully automated GeneXpert testing device, required minimal training and biosafety considerations. A total of 1,302 HIV VL, 277 EID and 1,581 MTB/RIF samples were tested on a four module GeneXpert platform in each study site. Xpert HIV-1 VL testing was prioritized for patients who presented with advanced HIV disease, pregnant women, adolescents and suspected ART failures patients. On average, the study sites had a GeneXpert utilization rate of 50.4% (Gutu Mission Hospital), 63.5% (Murambinda Mission Hospital) and 17.5% (Chimombe Rural Health Centre) per month. GeneXpert polyvalent testing error rates remained lower than 4% in all sites. Decentralized EID and VL testing on Xpert had shorter overall median TAT (1 day [IQR: 0–4] and 1 day [IQR: 0–1] respectively) compared to centralized testing (17 days [IQR: 13–21] and 26 days [IQR: 23–32] respectively). Among patients with VL >1000 copies/ml (73/640; 11.4%) at GMH health facility, median time to enhanced adherence counselling was 8 days and majority of those with documented outcomes had re-suppressed VL (20/32; 62.5%). Median time to ART initiation among Xpert EID positive infants at GMH was 1 day [IQR: 0–1].

**Conclusion:**

Implementation of near point-of-care GeneXpert platform for integrated multi-disease testing within district and sub-district healthcare settings is feasible and will increase access to VL, and EID testing to priority populations. Quality management systems including monitoring of performance indicators, together with regular on-site supervision are crucial, and near-POC test results must be promptly actioned-on by clinicians for patient management.

## Introduction

Despite significant increased access to antiretroviral therapy (ART), only 19 million HIV infected people are on treatment from an estimated 37 million people infected with HIV globally, and despite a substantial decline in AIDS-related deaths, 1 million people are still dying every year [1]. Access to early infant diagnosis (EID) for HIV-exposed infants has improved, however, in priority countries, only 50% received EID test in the first 2 months [2] and rapid diagnosis is critical for reducing mortality, which is highest at 2–3 months of age [3]. In a point-of-care (POC) EID trial, Jani I *et al* showed that the median time to EID result delivery from centralized testing was 125 days in Mozambique [4]. Intensified efforts to virtually eliminate transmission of mother to child infection coupled with earlier diagnosis are needed.

World Health Organization (WHO) recommends the use of Viral Load (VL) for monitoring ART [5], consequently, the VL testing unmet need is increasing in low-resource high-burden settings, as more people are initiated on ART with the ‘test and treat’ approach [6].

Tuberculosis (TB) remains among the common cause of illness and death amongst people living with HIV of all ages, causing about one third of AIDS-related deaths in 2015 [2]. In 2013, the global estimate for TB prevalence was 11 million; the majority of prevalent cases (81%) were reported in the 22 countries classified by the WHO as ‘high burden’ [2, 7]. WHO End TB Strategy targets to reduce TB related deaths by 95% and new cases by 90% by 2035, with the ultimate goal of ending the TB epidemic [7]. Successful implementation of ART and anti-tuberculosis therapy is dependent on the ability to diagnose, treat and monitor both infections.

The need to diagnose HIV and TB, together with high rates of loss to follow up and poor retention in care, influence the urge to decentralize laboratory services [8–11]. This process is limited by lack of laboratory infrastructure, technical skill and poor integration of HIV and TB services [7]. POC technology has been successfully employed in previously laboratory-based tests like CD4 testing [12] resulting in increased ART initiations and improved retention-in-care [13].

Contributions from governments, international non-governmental organizations and public/private partnerships have improved access to the GeneXpert platform following the WHO recommendation for its use in 2010[14] and it has transformed TB testing globally. In 2015, Cepheid (Cepheid Inc. Sunnyvale CA, USA) launched the GeneXpert^®^ HIV-1 VL (Xpert VL) and GeneXpert^®^ HIV-1 Qual (Xpert EID) assays for measuring HIV-1 VL in plasma and detecting HIV-1 in dried blood spots (DBS) or whole blood samples, respectively, and both assays have been pre-qualified by WHO [15]. The diagnostic accuracy of Xpert VL and Xpert EID tests has been shown to be comparable to reference testing assays [16]. Access to routine VL and EID remain restricted in low-income countries [17, 18] and the GeneXpert polyvalent testing platform, holds the potential to expand the roll-out of VL and EID to patients at high risk of morbidity and mortality.

Currently, Zimbabwe has about 135 GeneXpert platforms in more than 100 public health facilities and they are utilized solely for MTB/RIF testing. Medecins Sans Frontières’ (MSF) programmatic data and literature reviews have shown that GeneXpert capacity is underutilized [19] and there’s a need to leverage from these existing GeneXpert platforms to expand access to HIV VL and EID testing. This study sought to evaluate the operational feasibility of implementing the polyvalent Xpert system (Xpert HIV-1 VL, Xpert HIV-1 Qual and Xpert MTB/RIF assays) in decentralized district and sub-district in Zimbabwe.

## Methods

### Study sites

This was a prospective field feasibility evaluation study, carried out in rural Zimbabwe from November 2015 to August 2016. The diagnostic validation was completed at National Microbiology Reference Laboratory (NMRL), Harare, Zimbabwe. The feasibility evaluation was carried out at three sites: Gutu Mission hospital (GMH) laboratory (district laboratory), Murambinda Mission hospital (MMH) laboratory (district referral laboratory) and Chimombe Rural Health Clinic (CRHC), a primary health care facility. Study sites were selected within districts with MSF HIV/TB operational programs guided by moderate/high sample volumes (≥500 annual HIV VL referrals, ≥100 HIV EID referrals and ≥200 TB suspects from district hospital and ≥250 annual VL and 100 TB suspects from sub-district health center), and testing coverage gaps. CRHC also acted as ‘near-POC hub’ and provided laboratory-testing services for nearby health clinics in its catchment area (Mazuru, Nemashakwe, Munyikwa, Chepiri and Matarutse).

### Study population

Consenting HIV-positive patients, having venous blood taken for VL testing as part of their standard clinical management in accordance with the national VL testing algorithm [20] were recruited. Specimens were obtained prospectively from patients who have been on ART for at least 3 months, aged 18 years and above. For EID, HIV-exposed infants, aged six weeks to eighteen months, due for EID testing in accordance with the national EID algorithm [20] were recruited. Sputum samples were collected from any patient, suspected of TB and all study participants (or their guardians) provided written informed consent (or finger print for illiterate participants). Ethics approval was obtained from the Medical Research Council of Zimbabwe (MRCZ/A/1925) and Medecins Sans Frontières Ethics Review Board (ID 1504).

### System adaptation and user training

Prior to installation of the GeneXpert platforms in the three study sites, laboratory refurbishments were done which included, installation of air conditioning units, power back-up, refrigerators to store samples, dust control and security upgrades. Four modular Xpert devices including thermomixer, smart-block and a centrifuge, were installed in each study site. The district laboratories had biosafety cabinets (BSC) where sputum manipulations were done before testing in the GeneXpert. The microscopy site (CRHC) had one room that was used for sputum smear staining, microscopy and the GeneXpert was placed inside this air-conditioned room. There was no BSC and sputum manipulations (sputum smearing for microscopy and sputum prep for Xpert MTB/RIF) were done outside the CRHC microscopy lab, below an existing small shade.

The staff involved at all the 3 study sites (6 microscopists and 4 lab techs) had a 2-day training on the operation of the Xpert platform and a post-training theoretical competency assessment, based on general instrument usage and on comparison of testing results from in-house testing panels (known positive and negative samples). Standard operating procedures (SOPs) were developed and quality of testing was assessed by relying on internal Quality Controls (QC) provided for in the GeneXpert reagent cartridges (Sample Processing Control and Probe Check Control), use of in-house prepared controls and following basic Good Clinical Laboratory Practice (GCLP). Regular monitoring and on-site supervisions were conducted to improve the quality of testing, instrument maintenance, troubleshooting, assessing communication of results for linkage to care and monitoring of testing error rates.

### Sample collection and laboratory procedures

The health facility nurse drew whole blood into a standard 4ml K_2_EDTA tube (BD Vacutainer; Becton Dickinson Vacutainer Systems, New Jersey, USA) through phlebotomy for Xpert HIV-1 VL; while heel prick blood, for Xpert HIV-1 Qual test, was collected (50–70 μL) on a Whatman protein saver card 903 (Fischer Scientific, New Hampshire, USA). K_2_EDTA whole blood samples were taken to the facility laboratory within six hours of collection, where they were first spotted on a DBS card for centralized VL testing and then the remaining K_2_EDTA blood sample was centrifuged immediately at 1600g for 20 minutes. Using a transfer pipette, 1 mL of the plasma sample was transferred into a patient-ID-labeled Xpert HIV-1 Quant reagent cartridge and loaded into the Xpert instrument for VL testing.

For HIV EID testing, one DBS circle was excised from each patient sample using a sterile pipette tip and transferred into a 1 mL Xpert HIV-1 Qual assay sample reagent vial and incubated at 56°C in a thermomixer set at 500 rpm for 15 minutes. All the liquid from the lysed DBS specimen in the vial was then transferred into a patient-ID-labelled Xpert HIV-1 Qual cartridge and loaded into the Xpert instrument.

For Xpert MTB/RIF, the sample reagent was added into the sputum container (2:1 v/v), vigorously mixed and incubated for 15 minutes at room temperature. The liquefied sample was added into the Xpert MTB/RIF cartridge and loaded into the Xpert instrument. All TB suspect sputum samples were processed in the Xpert platform within the facility laboratory, as well as sputum smear negative samples (light microscope). All the specimen processing was performed as per manufacturer’s instructions [15]. Standard Operating Procedures (SOPs) can be found in the supporting information section (S4, S5 and S6).

The Xpert system with four modules were each used at the study sites, and they automate and integrate nucleic acid extraction and amplification with the detection of the target sequence in real-time [15]. At the district hospital laboratories (GMH and MMH), the tests (VL, EID and MTB/RIF tests) were conducted by laboratory scientists/technicians and microscopist. At the microscopy rural health facility, (CRHC) the tests were conducted by a microscopist. All tests results were used for clinical management in all sites, except in MMH where the use of Xpert HIV-1 VL for patient management was not approved by local health authorities.

Used Xpert HIV-1 VL and Qual cartridges were sent for incineration once a month at a central hospital incinerator. There was neither instrument breakdowns nor module replacement during the feasibility study period.

Due to relatively high HIV VL testing volumes at the district laboratories (>80 monthly), only priority VL samples (pregnant women, adolescents, and suspected ART failure patients) were processed on the Xpert platforms (as per clinician’s request), together with all HIV EID and all TB suspect samples. VL for patients who are stable on ART, were sent for centralized testing as DBS samples.

After the decentralized testing, the DBS samples for HIV VL and for HIV EID were all sent for confirmatory testing at centralized laboratories (NMRL and Mutare Provincial Hospital laboratory) and tested on BioMerieoux NucliSENS EasyQ/EasyMag (Marcy I’Etoile, France) and on the Roche COBAS AmpliPrep COBAS TaqMan HIV-1 Qualitative (Basel, Switzerland) respectively.

Data on GeneXpert polyvalent testing results, patient outcomes, including infrastructural and logistical requirements, were collected. Simple descriptive statistics was conducted and reported. The study was conducted over a 10-month period.

## Results

### Characteristics of patients enrolled into the study

Between November 2015 and August 2016, a total of 1,302 HIV VL, 277 EID and 1,581 MTB/RIF tests were conducted on Xpert systems at the three study sites. The median age of the Xpert HIV-1 VL study participants was 40 years [IQR: 33–49] and for Xpert HIV-1 Qual, it was 6.9 weeks [IQR: 6.1–9.1] and for Xpert MTB/RIF, was 40 years [IQR: 33–48] (Table 1). Study data can be found in the supporting information section (S1, S2 and S3).

**Table 1:** Characteristics of patients enrolled into the study across the three sites.

	VL	EID	TB	Total tested samples
**Health facility**				
GMH (district lab)	640	139	438	1,217
MMH (district referral lab)	417	80	1,028	1,525
CRHC (microscopy site)	245	58	115	418
**Gender**				
Male	460 (35.3)	128 (46.2)	544 (34.4)	1,132
Female	823 (63.2)	124 (44.8)	962 (60.8)	1,909
Unknown	19 (1.5)	25 (9.0)	75 (4.7)	119
**Median age for Xpert HIV-1 VL (years) [IQR]**	40 [33–49]	-	-	-
**Median age for Xpert HIV-1 Qual (weeks) [IQR]**	-	6,9 [6.1–9.1]	-	-
**Median age for Xpert MTB/RIF (years) [IQR]**	-	-	40 [33–48]	-

Key: **GMH**-Gutu Mission Hospital; **CRHC**-Chimombe Rural Health Centre, **MMH**-Murambinda Mission Hospital

### GeneXpert polyvalent testing

High levels of proficiency among all trained staff were observed and after conducting a median of 5 EID and 3 VL tests, the users felt comfortable to carry out the test independently. Prior knowledge of computers quickened the speed of attaining competency to operate the GeneXpert platform. The stringent sample storage and transport requirements for plasma/whole blood for HIV VL testing limited possible VL referral from the spoke sites to the CRHC testing hub, and only sputum and DBS EID samples could be referred, however, regular transport was not always available. Dust seepage into the laboratories and high summer temperatures (40°C) necessitated infrastructural re-enforcements (window/door seals).

GMH spent on average, 121 test cartridges per month whereas MMH used 153 cartridges while CRCH used about 42 cartridges per month on one 4 modular Xpert and the utilization rates were 50.4%, 63.5% and 17.5% respectively (Table 2).

**Table 2:** Polyvalent testing (HIV-1 EID, HIV-1 VL and MTB/RIF) profiles at the three study sites.

	GMH	MMH	CRHC
**Decentralized Xpert HIV-1 Qual**			
Total tested	139	80	58
Positivity rate	2.20%	2.50%	12.10%
Error rate	2.90%	1.40%	3.40%
^¤^Median days to result delivery to clinician [IQR]	1 [0–3]	8 [6–12]	1 [0–1]
Result concordance with centralized EID testing	100%	100%	98.20%
Distance from centralized lab	275km	180km	300km
**Conventional centralized EID testing**			
Median days to result delivery to clinician [IQR]	14 [12–16]	21 [17–30]	20 [17–23]
**Decentralized Xpert HIV-1 Quant**			
Total tested	640	417	245
§Viral load detectability rate	11.40%	5.90%	9.30%
Error rate	3.70%	3.60%	3.70%
Median days to result delivery clinician [IQR]	1 [0–1]	1 [0–2]	0 [0–1]
*Result concordance with centralized VL testing	98.20%	98.50%	96.80%
Distance from centralized lab	240km	250km	265km
**Conventional centralized VL testing**			
Median days to result delivery to clinician [IQR]	24 [23–29]	27 [24–31]	34 [24–45]
**Xpert MTB/Rif**			
Total samples tested	438	1028	115
Positivity rate	10.60%	9.60%	11.30%
Rif positivity rate	0.50%	0.58%	0
Error rate	3.40%	1.90%	2.60%
Median days to result delivery to clinician [IQR]	1 [0–1]	1 [0–2]	0 [0–1]
**Average polyvalent cartridges spent per month on a 4 modular machine**	121	153	42
**GeneXpert utilization rate**¥	50.40%	63.50%	17.50%

^**§**^VL detectability rate = VL ≥1000 copies/mL

^**¤**^ Median days to results to the clinician at the facility with the GeneXpert instrument

*Result concordance estimated at the 1000 copies/mL threshold

^**¥**^ Estimates based on calculations that one 4-module GeneXpert device has capacity to process 3,000 tests per year.

GMH-district lab; MMH-district referral lab, CRHC-Microscopy site lab

Decentralized EID and VL testing on Xpert had the shortest overall median turn-around time (TAT) for result delivery to the clinician (1day for both) compared to centralized testing (17 days and 26 days, respectively), (Table 2). The intention was to provide results within the same day whilst the patient is still on triage within the study facility.

The Xpert HIV-1 VL and Qual testing error rates were comparable among the study sites (Table 2) and the most common error was sample volume insufficiency, whilst for Xpert MTB/RIF, it was sputum viscosity and/volume.

The GeneXpert system proved easy-to-use for polyvalent testing, with results accessible within 90 minutes for VL and EID whereas MTB/RIF was within 120 minutes. DBS sample preparation for EID testing (sample elution) took an average of 20 minutes (5 minutes hands-on time and 15 minutes thermomixer incubation) whereas plasma sample preparation for VL took 23 minutes (20 minutes for sample centrifugation and 3 minutes hands-on time). During polyvalent testing, Xpert cartridges with samples can be continuously loaded into the Xpert instrument when there are free modules. A routine workflow was implemented and it included separate work areas for preparing DBS EID and plasma VL for Xpert polyvalent testing.

The addition of the near-POC instrument was not an extra burden for laboratory staff in all the 3 study sites as anonymous self-completed interview questionnaires reported high levels of staff satisfaction with the instrument.

### Outcomes from decentralized GeneXpert polyvalent testing

The VL detectability (VL>1000copies/ml) was low (Table 3). In limiting the analysis to those individuals eligible for Enhanced Adherence Counselling (EAC), who had data available in the EAC registers, their median time to EAC was 23 days at CRHC and 8 days at GMH (Table 3).

**Table 3:** Outcomes for patients tested on Xpert HIV-1 VL and HIV-1 Qual per facility.

	GMH	MMH	CRHC
**All individuals with VL >1000**	73 (11.4%)	25 (5.9%)	23 (9.3%)
Median time to EAC initiation ɠ	8 days [IQR: 5–14]	§	23 days [IQR:12–33]
EAC outcome*			
VL re-suppressed	20 (27.4%)		4 (17.5%)
VL un-suppressed	6 (8.2%)	§	3 (13%)
Switched to second line	6 (8.2%)		4 (17.5%)
No outcome documented	41 (56.2%)		12 (52%)
**Infants with positive EID**	3	2	7
Median time to ART initiation (days) ɠ	1 [0–1]	§	2 [1–3]

^**ɠ**^ Median time only estimated for patients presenting at health facilities with *on-site* GeneXpert platform (hubs)

*Abstracted from the EAC registers in study facilities

^§^ The results in this facility were never used for patient management as use of GeneXpert results for patient management was not approved by district health authorities.

GMH-district lab; MMH-district referral lab, CRHC-Microscopy site lab

In GMH and CRHC, majority of those individuals with documented EAC outcomes, had a re-suppressed VL (20/32; 62.5% and 4/11; 36.4% respectively) (Table 3). Among patients with documented EAC outcomes, a substantial proportion of patients with continued un-suppressed VL were switched to 2^nd^ line ART in GMH and CRHC (6/32; 18.8% and 4/11; 36.4%) respectively while, all the EID positive children at these two sites, were immediately commenced on treatment near or same day.

## Discussion

This study showed that placement and polyvalent testing with the GeneXpert platform was possible at a microscopy site and in district hospital laboratories; however, additional resources and adaptation of laboratory and clinic work-flow procedures are required. For facilities with already-existing GeneXpert instruments for MTB/RIF diagnosis, possible required upgrades to enable polyvalent testing may include: GeneXpert software upgrade to 4.6 or newer versions, refrigerators for plasma sample storage and or cartridges, centrifuge and smartblock with thermomixer. To maximize patient outcomes and cost effectiveness, placement of the GeneXpert near POC platform must consider testing volumes, reliable sample/result transport systems, existing laboratory network and human resource capacity. The testing was easily task-shifted to microscopists, as they already had prior exposure to MTB/RIF testing. Even though anonymous self-completed interview questionnaires showed high laboratory staff satisfaction levels with the GeneXpert device implementation, it is critical to ensure adequate human resources to minimize overburdening of laboratory staff.

PCR is extremely sensitive and thus poses a high risk of contamination [21, 22], which was experienced during this study due to prior limited GCLP at the microscopy site (CRHC). Plasma VL sample contaminated a DBS EID sample resulting in a false positive EID result and it is advisable to have separate work-stations for EID and VL sample preparation in addition to clearly defined workflows, and GCLP standards, so as to ensure accurate and reliable near-POC testing results. Regular on-site supervision must be an on-going exercise including quality assessments through either external or internal quality control programs [21]. Furthermore, GeneXpert systems have the ability for remote connectivity from different providers (C360 from Cepheid, GxAlert from SystemOne, DataToCare form Savics, among others [23]) which could help to facilitate trouble-shooting, provision of pro-active maintenance, as well as testing data transmission to program managers’ dashboards.

In the present study, GeneXpert testing was prioritized for all HIV-exposed infants for EID and all samples from presumptive TB patients whereas for HIV VL, priority was given to patients who presented with advanced HIV disease, pregnant women, adolescents, suspected ART failures and defaulter patients. HIV VL for patients who are stable on ART, were sent for centralized testing as DBS samples. Such approaches of differentiated care will help reduce the heightened risk of severe morbidity and mortality especially among these priority patients [24–26]. This prioritization procedure did not cause any considerable interference to the TB testing patterns and the established maximum utilization capacities of the devices were not reached. Nonetheless, significant volumes of HIV testing cartridges could possibly overload existing devices and comprehensive site mapping together with prioritization of testing for critical populations could ensure a phased implementation of polyvalent testing. Moreover, there’s need to improve in-country collaborations between National TB and HIV/AIDS programmes as this still hinders leveraging of existing limited resources. Collaboration between programmes could also help to establish cooperation in provision of service and maintenance, human resources, supply chain management and even in set-up costs.

In this study, decentralized Xpert HIV-1 VL testing was more beneficial for patients who presented at the study health facilities rather than its use in a spoke and hub that normally had transport challenges. The lack of efficient and robust specimen referral networks will limit access to near-POC diagnostics, which require plasma for VL testing. In many settings, specimen referral transport networks are unnecessarily siloed and inefficient. This underscores the need for near-POC technologies that can also utilize DBS samples or innovative instrument-free plasma separators based on lateral flow filtration, which are suitable for storage and transportation at ambient temperature [27]. Furthermore, innovative sample transport systems which include the Uganda motorcycle sample collection project, use of postal services, use of SMS printers, mobile SMS among other alternative options for faster result delivery [28–31] must be considered so as to increase access to molecular testing hubs in districts or sub-district health facilities.

Compared to centralized testing, decentralized testing on Xpert had considerably shorter overall median TAT to result delivery (reduced from 27 days to 1 day for HIV VL and from 17 days to 1 day for HIV EID) which presented a shorter time to clinician action. Such a finding is in line with other studies that have shown that POC CD4 was also effective at reducing TAT to results and subsequently reducing time to ART initiation and patient loss to follow-up [12, 13, 32]. However, in our study, MMH facility had a higher median TAT to EID result delivery to clinicians (8days), probably because use of GeneXpert results for patient management was not approved by district health authorities in this site. Nevertheless, given that infant mortality is highest at 2–3 months of age, rapid diagnosis is critical to prevent illness and death [3]. A recent POC EID trial showed that infants in facilities with POC test devices, were seven times more likely to commence ART within two months as compared to conventional standard of care [3]. However, a recent randomized controlled trial in South Africa found that while POC led to a shorter time to ART initiation, loss to follow-up was higher in the POC arm and retention in care at 6 and 12 months was similar in the POC and standard-of-care arm [33]. This study highlights the need for other interventions to improve retention in care.

Median time to EAC for participants with unsuppressed VL in this study, remained high at CRHC (23 days [IQR: 12–33]) and introduction of near-POC platforms must be complemented with a strengthened health system in order to reduce any post-testing delays on clinicians acting on the patient results [34, 35]. Nonetheless, the VL detectability in this study population was low owing to the successfully implemented differentiated models of HIV care among other packages for improving patient outcomes [36, 37].

Of the 12 infants who tested positive on Xpert HIV-1 Qual, 10 (83.3%) were immediately initiated on ART; whereas those who tested HIV negative on Xpert HIV-1 Qual, had scheduled follow-up for HIV re-testing using RDTs to rule out any HIV infection [5]. Having polyvalent testing platforms available near-POC for patient management, could allow tests to be completed within a single clinical encounter with immediate issuance of results which could potentially inform important and urgent clinical decision making [4, 11], especially for patients at high morbidity and mortality risk. WHO has provided considerations for adoption and use of multidisease testing devices [38], however, pre-qualification diagnostic evaluations from WHO for multidisease testing platforms must be expedited for new tests to reduce time to market.

As substantial gaps exist, especially in Sub Saharan Africa, in the un-met need for HIV VL, EID and TB testing, and as resources continue to be scarce; polyvalent near POC diagnostics like Xpert (including the up-coming portable battery operated true POC, Xpert Omni [39]), can help integrate HIV and TB services into a single one-stop facility without the need for major restructuring of district facilities. However, these near-POC systems and related service and maintenance costs need to be accessible at reasonable prices, adapted to specific country contexts, and integrated effectively into the national laboratory networks.

Xpert HIV-1 VL and Qual reagent cartridge chambers (just like majority of VL/EID testing reagents from other platforms) [40–42] contain a chemical compound (Guanidine thiocyanate), used as a general protein denaturant and for the extraction of DNA and RNA [15]. This compound is highly toxic both to aquatic life and humans and must be incinerated at a high temperature (≥ 850°C) within the second combustion chamber with a retention time of 2 seconds [43, 44]. However, many healthcare facilities in need of these polyvalent platforms do not have incinerators whilst some have incinerators that do not reach these temperatures. International organizations, ministries of health, including reagent manufacturers, must ensure that funding is available for the construction of incineration technology together with policies and protocols for HIV molecular testing waste management. Cement factories can be an alternative to outsource incineration of Guanidine thiocyanate waste. Nevertheless, HIV molecular testing technologies which do not utilize Guanidine thiocyanate for the extraction of nucleic acids, must be considered.

The Xpert system had a relatively lower invalid result rate compared to other platforms [37, 45]. Most errors during Xpert HIV-1 VL testing were caused by plasma sample volume inadequacy as the required 1 mL of plasma proved to be difficult to obtain, especially with samples from children or patients with difficult veins. Moreover, it is worthwhile for the manufacturer to consider branding different cartridges with unique colour codes for different tests, so as to minimize mixing up of cartridges; especially as these are also used by less laboratory trained people. Even though the Xpert platform will alert the user of the wrong cartridge and test ordered, in the present study, this came too late after a patient sample, cartridge and time were wasted. In addition, the study encountered a few (3/277) Xpert HIV-1 Qual negative results which had PCR graphs with late amplifications (after 42 cycle thresholds) and this necessitated further internal reviews to evaluate if this was specific amplification. Such possibly ‘indeterminate’ results had to be confirmed by conventional centralized testing, however, the manufacturer asserts to have rectified this with an upgraded kit software assay definition file.

Nonetheless, inferences from this study should be made with caution as decentralization has its own limitations which include high cost and near-POC technology may not manage all district samples and there is a need to prioritize samples for decentralized testing. A cost-effectiveness study is currently under way to explore the cost of decentralized Xpert compared to centralized testing and also evaluating a potential cost sharing model between TB and HIV programs. We recommend future studies to assess the acceptability of integrated GeneXpert testing and impact of such a near POC testing system among priority patients.

Strengths of this study include the prospective multi-center design used in rural health facilities, large sample sizes and confirmatory testing for EID and VL. Despite same-day diagnosis, documentation of time to EAC and the outcomes of the EAC sessions need further investigations together with time to treatment initiation among TB patients.

### Conclusions

Findings from this study support feasibility of integrated testing (HIV VL, EID, and TB) in the GeneXpert near POC instrument within district and sub-district health facilities. Testing of priority VL samples, together with all EID and all TB suspect samples in new or existing GeneXpert excess capacity will benefit patients at most need, together with the TB and HIV programs. However, progressive collaborations between national HIV and TB programmes is crucial for integrated planning to enable effective utilization of these devises. In addition, multi-disease testing in new devices or existing devices, must be informed by estimated testing volumes across the diseases. Quality management systems including monitoring of performance indicators, together with regular on-site supervision are crucial and near-POC test results must be actioned-on by clinicians for patient management.

## Supporting information

S1 Data (XLSX)

S2 Data (XLSX)

S3 Data (XLSX)

S1 Text (PDF)

S2 Text (PDF)

S3 Text (PDF)
