# Supplementary material for: Multidisease testing for HIV and TB using the GeneXpert platform: A feasibility study in rural Zimbabwe
Source: PLoS One. 2018 Mar 2;13(3):e0193577. doi: 10.1371/journal.pone.0193577 (PMC5834185; doi:10.1371/journal.pone.0193577)
Supplement: S1 Text — (PDF) [file pone.0193577.s004.pdf]

Standard Operating Procedures for Quantitative HIV-1 viral load testing using GeneXpert technology

June 2015

VALIDATION

Table of Contents

I. Title of Procedure 3
II. Test Summary 3
III. Principle 3
IV. Specimen Handling and Preparation 4
V. Quality Control 4
VI. Reagents, Materials, & Equipment 6
VII. Procedure 7
VIII. Interpretation of Results 10
IX. Possible reasons for repeating the assay 13
X. Limitations 13
XI. Performance Characteristics 14
XII. References 15

Figure 1: Overview of the GeneXpert test cartridge 4
Figure 2: GeneXpert quality control result scenarios 6
Figure 3: GeneXpert transfer pipette and aerial view of the reaction cartridge 8
Figure 4: Summary of sample loading in GeneXpert HIV-1 quant 9
Figure 5: GeneXpert HIV-1 quantitative detectable VL 12
Figure 6: GeneXpert HIV-1 quantitative not detected VL 12

Table 1: GeneXpert HIV-1Viral load result interpretation 10

VALIDATION

I. Title of Procedure

Quantitative HIV-1 viral load testing using GeneXpert technology

II. Test Summary

State the physiologic and diagnostic reasons for performing the test.

Human Immunodeficiency Virus (HIV) is the etiologic agent of Acquired Immunodeficiency Syndrome (AIDS). It can be transmitted through sexual contact, exposure to infected blood or blood products, prenatal infection of a fetus, or perinatal or postnatal infection of a newborn. HIV diagnostics have evolved significantly in the past two decades and continue to be significantly important in saving lives of millions of HIV infected patients. Today, measurement of blood plasma HIV-1 RNA concentration known as viral load using nucleic acid-based molecular diagnostic assays has been established as standard of care in assessing HIV-positive patient prognosis and response to antiretroviral therapy. Untreated HIV-1 infection is characterized by high-level viral production and CD4 T-cell destruction, despite an often lengthy clinical latency, to significant net loss of CD4 T cells and AIDS. Assessment of viral load levels is a strong predictor of the rate of disease progression and, by itself or in combination with CD4T-cell counts, has great prognostic value. The HIV-1 Quant Assay, performed on GeneXpert® Instrument Systems, is designed for the rapid quantitation of Human Immunodeficiency Virus type 1 (HIV-1) in human plasma from HIV-1 infected individuals over the range of 40 to 10,000,000 copies/mL. HIV-1 Group M subtypes A, B, C, D, AE, F, G, H, AB, AG, J, K and Groups N and O are detected and quantified by the HIV-1 Quant Assay. The HIV-1 VL Assay uses reverse transcriptase polymerase chain reaction (RT-PCR) technology to achieve high sensitivity for the quantitative detection of HIV-1 RNA in human plasma from HIV-1 infected individuals.

III. Principle

GeneXpert Instrument Systems automate and integrate sample preparation, nucleic acid extraction and amplification, and detection of the target sequence in simple or complex samples using real-time reverse transcriptase PCR (RT-PCR). The systems consist of an instrument, personal computer, and preloaded software for running tests and viewing the results. The systems require the use of single-use disposable GeneXpert cartridges that hold the RT-PCR reagents and host the RT-PCR processes. Because the cartridges are self-contained, cross-contamination between samples is minimized. The HIV-1 VL Assay includes reagents for the detection of HIV-1 RNA in specimens and two internal controls used for quantitation of HIV-1 RNA. The internal controls are also used to monitor the presence of inhibitor(s) in the RT and PCR reactions. The Probe Check Control (PCC) verifies reagent rehydration, PCR tube filling in the cartridge, probe integrity, and dye stability.

VALIDATION

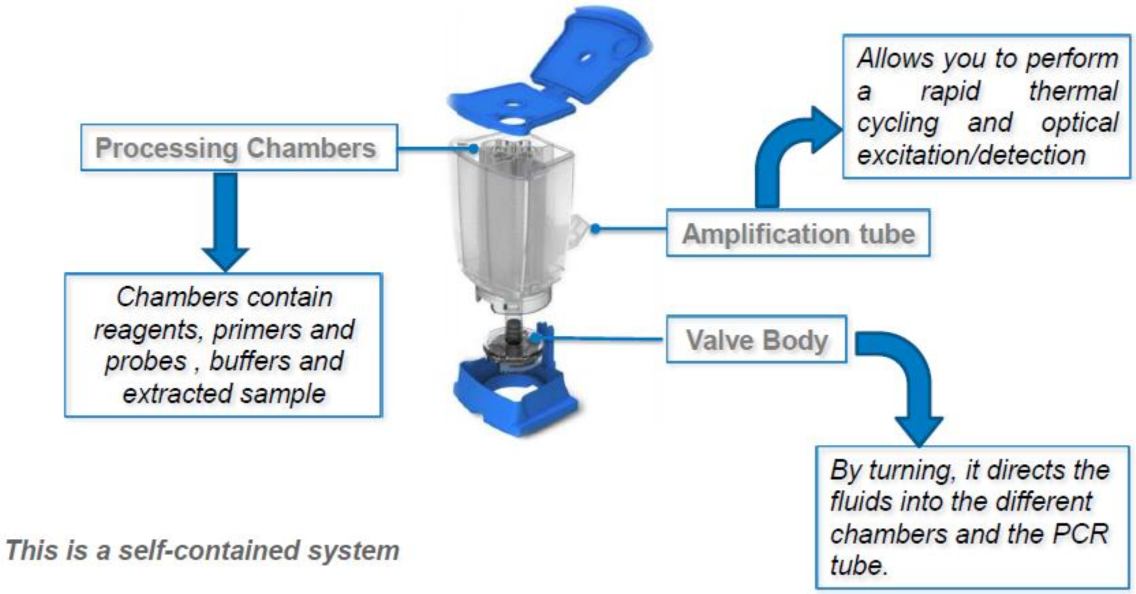

Figure 1: Overview of the GeneXpert test cartridge

IV. Specimen Handling and Preparation

- Whole blood should be collected in EDTA, EDTA-PPT, or ACD collection tubes and centrifuged to separate the plasma and red blood cells per the manufacturer's instructions.
- A minimum of 1 mL plasma is required for the HIV-1 VL Assay. If using the transfer pipette included in the kit, a minimum of 1.2 mL plasma is required. Alternatively, if using a precision pipette, a minimum of 1 mL plasma is required.
- Whole blood may be held at 15–30 °C for up to 8 hours or at 2–8 °C for up to 72 hours, prior to preparing and testing the specimen. After centrifugation, plasma may be held at 15–30 °C for up to 24 hours or at 2–8 °C for up to 6 days, prior to testing.
- Plasma specimens are stable frozen (≤ -18 °C and ≤ -70 °C) for six weeks.
- Plasma specimens are stable up to three freeze/thaw cycles.
- Plasma specimens must be thawed and equilibrated to room temperature prior to transfer to cartridge.

V. Quality Control

Each GeneXpert test cartridge is a self-contained test device with an in-built control for each sample. Normally, no external controls are required. The internal controls enable the system to detect specific failure modes within each for each sample

- **Instrument system control**: Check status-it checks the optics, temperature of the module and the mechanical integrity of each cartridge. If the system controls fail, an ERROR test result will be reported.

- **Probe Check control (PCC)**: after sample preparation, bead reconstitution and tube filling (prior to thermal cycling), multiple fluorescent readings are taken at different temperatures and compared to default setting. PCC controls for:
    - Missing target specific reagent (TSR) and or enzyme reagent beads which contain all primers, probes and internal control template.
    - Incomplete reagent reconstitution
    - Incomplete reaction tube filling
    - Probe degradation

  If the PCC fails, an ERROR test result will be reported.

- **Sample processing control (SPC)** assesses the effectiveness of the sample processing steps, including and up-to reaction tube filling. SPC ensures that the sample was correctly added to the cartridge and detects degradation of the enzyme(s) or other components of the system. SPC does not compete with target DNA.
    - SPC must be Positive when target is Negative
    - SPC can be Positive or Negative when the target is Positive
    - SPC passes if it meets the validated acceptance criteria. E2097: too less sample volume added (<1mL), E2096: no sample added

- **Internal Quantitative Standard High and Low (IQS-H and IQS-L)**: IQS-H and IQS-L are two dry bead armored RNAs nonspecific to HIV in the form of a dry bead that goes through the whole GX process. The IQS-H and IQS-L are standards calibrated against the WHO 3rd International Standard. They are used for quantification by using lot specific parameters for the calculation of HIV-1 RNA concentration in the sample. The IQS-H and IQS-L pass if they meet the validated acceptance criteria. They run internally with every cartridge and they control for reagent performance due to improper storage and they confirm that reaction components are set up correctly.
- **External Controls**: not available in the kit, but they can be used (positive and negative controls).

VALIDATION

Xpert Assay and System Control
Summary

Procedural Step & Condition Controlled		IQS	Probe Check	System Checks	Final Results
Pre-, and during run	Heating, cooling, pressure (clogging), electronics, optics			X	Failure results in: ERROR
Sample Addition	Sample aspiration			X	Failure results in: ERROR
Sample Processing	Cell lysis	X			Failure results in: ERROR
	Nucleic acid recovery	X			Failure results in: ERROR
Reagent Addition	Bead Presence/Rehydration	X	X		Failure results in: ERROR or INVALID
	PCR tube fill	X	X		Failure results in: ERROR
	Fluorogenic detection	X	X		Failure results in: ERROR
PCR	Thermal cycling	X		X	Failure results in: ERROR
	Primer or target annealing	X			Failure results in: INVALID
	Inhibition	X			Failure results in: INVALID
Quantitation	Results Reported	X			Quantitative Value (IU/ml or copies /ml)<br>Detected Above range<br>Detected Below Range<br>Not Detected

Figure 2: GeneXpert quality control result scenarios

VI. Reagents, Materials, & Equipment

The HIV-1 VL Assay kit contains sufficient reagents to process 10 specimens or quality control samples. The kit contains the following:

1. 10 HIV-1 VL Assay Cartridges with Integrated Reaction Tubes. Each cartridge contains:
    - Bead 1, Bead 2, and Bead 3 (freeze-dried)
    - Lysis Reagent (Guanidinium Thiocyanate) 2.0 mL
    - Rinse Reagent 0.5 mL
    - Elution Reagent 1.5 mL
    - Binding Reagent 2.4 mL
    - Proteinase K Reagent 0.48 mL
2. Disposable 1 mL Transfer Pipettes 10 per kit
3. CD 1 per kit
    - Assay Definition File (ADF)
    - Instructions to import ADF into GeneXpert software
    - Package Insert

Storage and Handling

- Store the HIV-1 VL Assay cartridges at 2–8 °C.
- Do not open the cartridge lid until you are ready to perform the test.
- Use cartridge within four hours after opening the cartridge lid.
- Do not use a cartridge that has leaked.

Materials Required but Not Provided

- Printer
- Bleach

Warnings and Precautions

- Treat all biological specimens, including used cartridges, as if capable of transmitting infectious agents. Because it is often impossible to know which might be infectious, all biological specimens should be treated with standard precautions.
- Do not open the HIV-1 VL Assay cartridge lid until you are ready to add the plasma specimen
- Do not use a cartridge that has been dropped after removing it from the packaging.
- Do not shake the cartridge. Shaking or dropping the cartridge after opening the lid may yield invalid results.
- Do not place the sample ID label on the cartridge lid or on the barcode label.
- Each single-use HIV-1 VL Assay cartridge is used to process one specimen. Do not reuse spent cartridges.
- Do not use a cartridge that has a damaged reaction tube.
- Single-use disposable pipette is used to transfer one specimen. Do not reuse disposable pipettes.
- Wear clean lab coats and gloves. Change gloves between processing each sample.
- In the event of contamination of the work area or equipment with samples or controls, thoroughly clean the contaminated area with a solution of 1:10 dilution of household chlorine bleach and then 70% ethanol. Wipe work surfaces dry completely before proceeding.

VII. Procedure

1. Preparing the Specimen

- If using frozen specimens, place the specimens at room temperature 20–35 °C until completely thawed and equibrate to room temperature before use.
- Plasma samples stored in 2–8 °C should be removed from the refrigerator and equilibrated to room temperature before use.
- Vortex plasma for 15 seconds before use. If the specimen is cloudy, clarify by a quick spin.

2. Preparing the Cartridge

- Wear protective disposable gloves.
- Inspect the test cartridge for damage. If damaged, do not use it.
- Open the lid of the test cartridge.
- Allow samples to come to room temperature prior to loading plasma into the cartridge

• **Option 1:** If using the transfer pipette included in the kit (Figure 1), fill to just below the bulb but above the line to transfer at least 1 mL plasma from the collection tube into the sample chamber of the test cartridge (Figure 2). Do **NOT** pour the specimen into the chamber!

VALIDATION

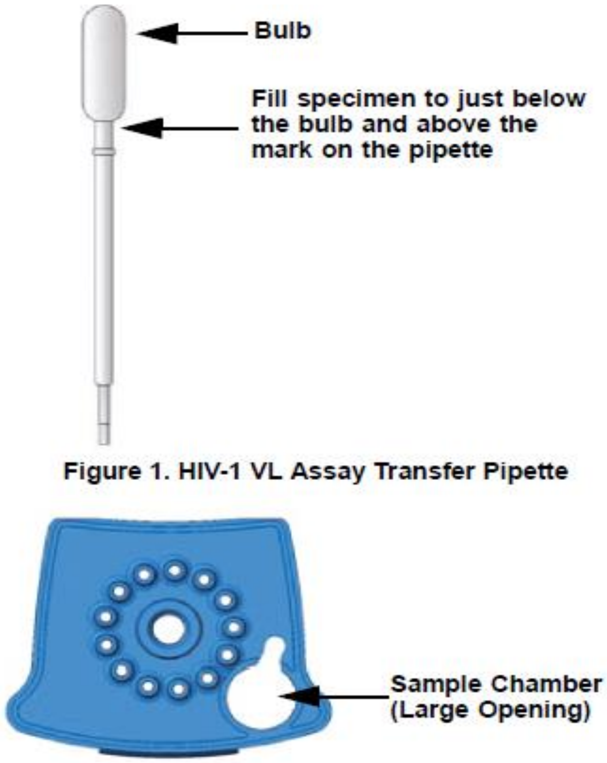

Figure 3: GeneXpert transfer pipette and aerial view of the reaction cartridge

- Close the cartridge lid.
- Load the cartridge into the GeneXpert instrument or Infinity system.

Note There is a thin plastic film that covers the inner ring of 13 ports of the test cartridge. This film should not be removed.

Option 2: If using an automatic pipette, transfer at least 1 mL of plasma into the sample chamber of the test cartridge (Figure xx). Do **NOT** pour the specimen into the chamber!

Important Start the test within four hours of adding the sample to the cartridge

Loading less than 1 mL plasma into the cartridge will trigger an insufficient volume error (ERROR 2097), preventing the instrument from running the sample

Starting the Test

This section lists the basic steps for running the test. For detailed instructions, see the GeneXpert System Operator Manual on the computer desktop.

a. Turn on the GeneXpert instrument. First turn on the instrument and then turn on the computer. The GeneXpert software will launch automatically. If it doesn't, double-click the GeneXpert
b. Software shortcut icon on the Windows® desktop.

VALIDA

c. Log on to the GeneXpert Instrument System software using your user name and password. Click Create Test
d. Scan in the Patient ID (optional). If typing the Patient ID, make sure the Patient ID is typed correctly. The Patient ID is associated with the test results and is shown in the View Results window.
e. Scan or type in the Sample ID. If typing the Sample ID, make sure the Sample ID is typed correctly. The Sample ID is associated with the test results and is shown in the View Results window and all reports. The Scan Cartridge dialog box appears.
f. Scan the barcode on the Xpert® HIV-1 Viral Load cartridge. The Create Test window appears. Using the barcode information, the software automatically fills the boxes for the following fields: Select Assay, Reagent Lot ID, Cartridge SN, and Expiration Date.
g. Click Start Test. Enter your password, if requested.
h. Open the instrument module door with the blinking green light and load the cartridge.
    - Close the door. The test starts and the green light stops blinking. When the test is finished, the light turns off.
    - Wait until the system releases the door lock before opening the module door and removing the cartridge.
    - The used cartridges should be disposed in the appropriate specimen waste containers according to your institution's standard practices.

TION

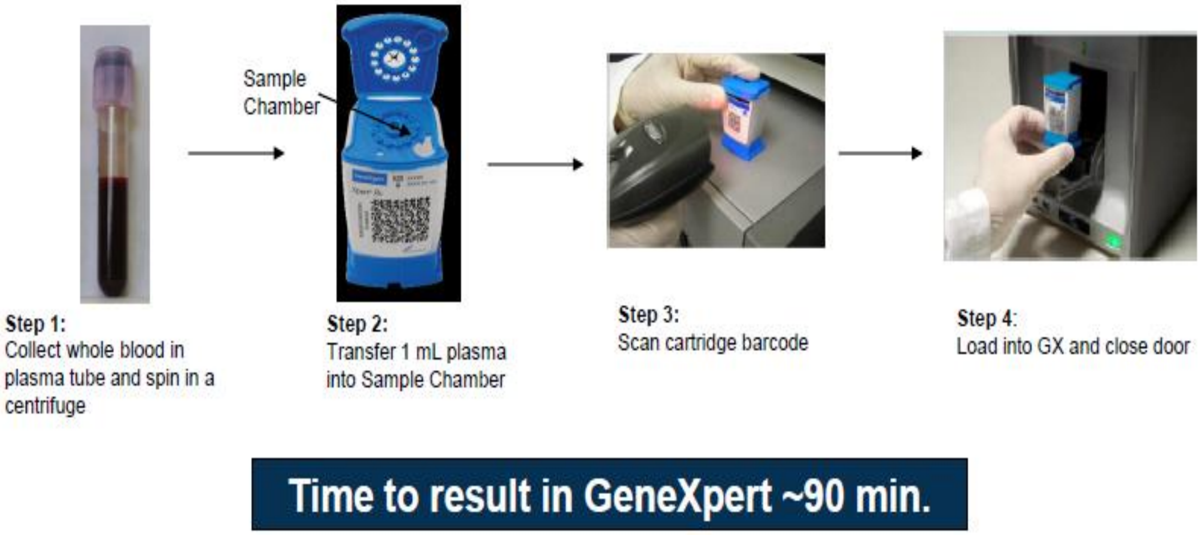

Figure 4: Summary of sample loading in GeneXpert HIV-1 quant

- **WARNING: GeneXpert HIV Quant cartridges contain a highly toxic chemical (to human and environment), Guanidinium Thiocyanate, and should be disposed of after use, in an incinerator capable of reaching 1200°C.**

VIII. Interpretation of Results

Click the View Results icon to view results.

Upon completion of the test, click the Report button of the View Results window to view and/or generate a PDF report file. The test results take approximately 90minutes to be produced

The results are interpreted automatically by the GeneXpert Instrument System from measured fluorescent signals and embedded calculation algorithms and are clearly shown in the View Results window (Figure 5 and Figure 6). Possible results are shown in Table 1.

VALIDATION

Table 1: GeneXpert HIV-1Viral load result interpretation

Result	Interpretation
**HIV-1 DETECTED**<br>**XX copies/mL**<br>See Figure 3.	HIV-1 RNA is detected at XX copies/mL.<br>• HIV-1 RNA has quantitative value within the analytical measurement range.<br>• IQS-H and IQS-L: PASS.<br>• Probe Check: PASS; all probe check results pass.
**HIV-1 DETECTED**<br>**> 1 × 10^7 copies/mL**	HIV-1 RNA is detected above the analytical measurement range.<br>• IQS-H and IQS-L: PASS.<br>• Probe Check: PASS; all probe check results pass.
**HIV-1 DETECTED**<br>**< 40 copies/mL**	HIV-1 RNA is detected below the analytical measurement range.<br>• IQS-H and IQS-L: PASS.<br>• Probe Check: PASS; all probe check results pass.
**HIV-1 NOT DETECTED**<br>See Figure 4.	HIV-1 RNA is not detected.<br>• IQS-H and IQS-L: PASS.<br>• Probe Check: PASS; all probe check results pass.
**INVALID**	Presence or absence of HIV-1 RNA cannot be determined. Repeat test according to the instructions in Section 15.2, Retest Procedure.<br>• IQS-H and/or IQS-L: FAIL; Cycle thresholds (Cts) are not within valid range.<br>• Probe Check: PASS; all probe check results pass.
**ERROR**	Presence or absence of HIV-1 RNA cannot be determined. Repeat test according to the instructions in Section 15.2, Retest Procedure.<br>• Probe Check: FAIL*; all or one of the probe check results fail.
**NO RESULT**	Presence or absence of HIV-1 RNA cannot be determined. Repeat test according to the instructions in Section 15.2, Retest Procedure. A **NO RESULT** indicates that insufficient data were collected. For example, the operator stopped a test that was in progress.

VALID

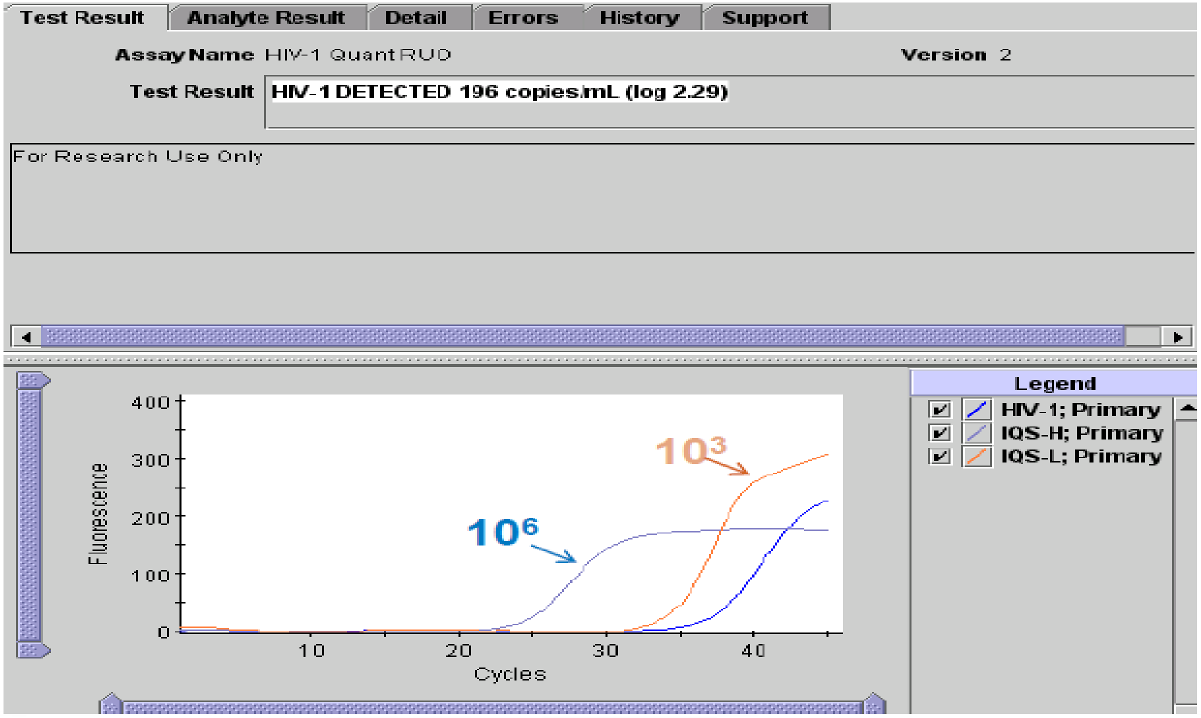

Figure 5: GeneXpert HIV-1 quantitative detectable VL

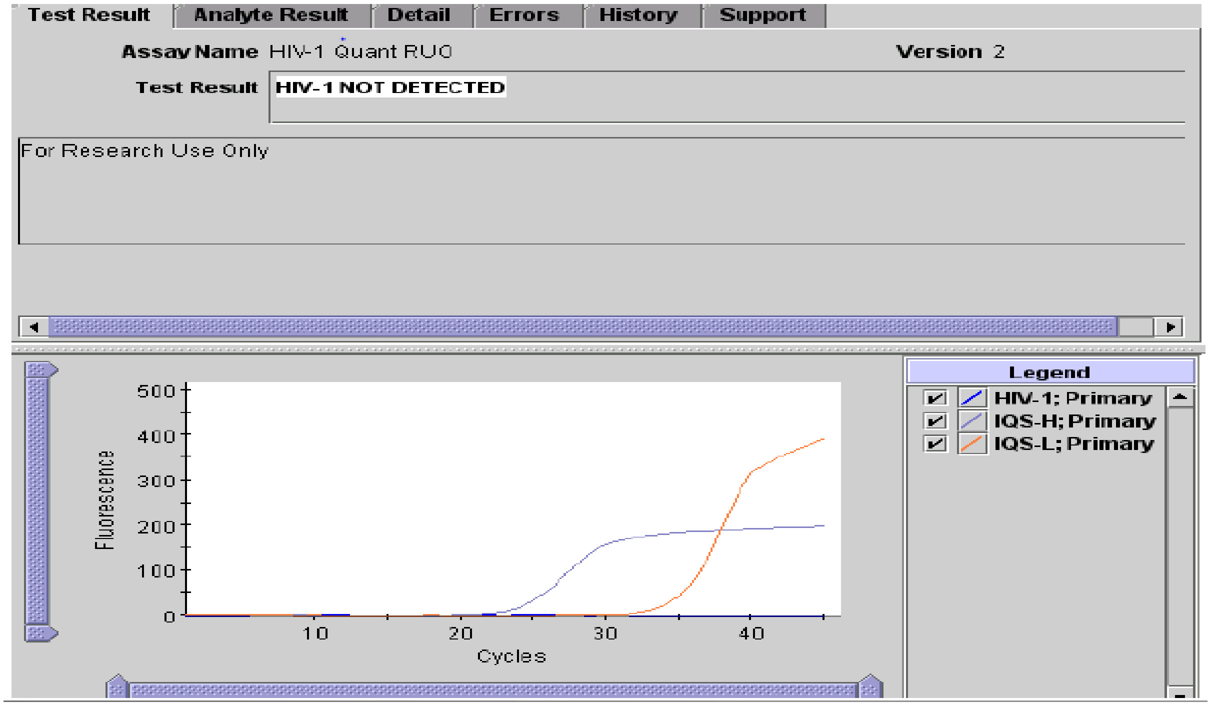

Figure 6: GeneXpert HIV-1 quantitative not detected VL

IX. Possible reasons for repeating the assay

•An INVALID result indicates that the sample was not properly processed, PCR was inhibited, or the sample was inadequate. An invalid result also indicates that the IQS-H and/or IQS-L Cts are not within valid range.

•An ERROR result indicates that the Probe Check Control failed or maximum pressure limits were exceeded. An error result indicates that the assay was aborted. Possible causes include: insufficient volume of sample was added, the reaction tube was filled improperly, a reagent probe integrity problem was detected, or the maximum pressure limit was exceeded.

•A NO RESULT indicates that insufficient data were collected. For example, the operator stopped a test that was in progress, a load error occurred, or the software was closed prematurely.

Retest Procedure

For retest of an **INVALID**, **ERROR**, or **NO RESULT**, use a new cartridge (do not re-use the cartridge).

1. Remove a new cartridge from the kit.
2. Section VII on preparing cartridge and starting the test on page 7.

X. Limitations

Good laboratory practices and changing gloves between handling specimens are recommended to avoid contamination of specimens or reagents.

Factors that negatively affect results

Interfering substance

–False negative test results or invalid results may be observed in the presence of interfering substance like; Albumin (90g/L), bilirubin (20mg/dL), haemoglobin (500mg/dL), human DNA (0,4mg/dL) and triglycerides (3000mg/dL).

Biological specimens, including used cartridges, should be treated as capable of transmitting infectious agents and should be discarded in hazardous clinical waste bins.

VALIDATION

XI. Performance Characteristics

Limit of Detection (LoD)
The LOD for the HIV-1 VL Assay is 40 cp/mL for HIV-1 subtype B in EDTA plamsa.

Limit of Quantitation
The LoQ is defined as the lowest concentration of HIV-1 RNA that is determined with acceptable precision and trueness. The LOQ of the HIV-1 VL Assay is 40 cp/mL (1.60 log10).

Linear Range
The HIV-1 VL Assay is linear within a range 30 to $1x10^{7}$cp/mL

Specificity
The specificity of the HIV-1 VL Assay was evaluated using EDTA plasma specimens from HIV-1 negative blood donors. The HIV-1 VL Assay specificity was demonstrated at 100% (95% CI = 96.7–100.0).

TECHNICAL SUPPORT
Raynolds Mangena
Pointe care diagnostics
Harare
+263 77 290 7343

Chepeid
South Africa
+27 11 234 9636
MOBILE +27 71 209 8069
FAX +27 11 234 9640
EMAIL dipti.lallubhai@cepheid.com

ATION

XII. References

1. Barre-Sinoussi F, Chermann JC, Rey F, et al. Isolation of a T-lymphotropic retrovirus from a patient at risk for acquired immune deficiency syndrome (AIDS). *Science* 1983;220:868–871.
2. Popovic M, Sarngadharan MG, Read E, et al. Detection, isolation and continuous production of cytopathic retroviruses (HTLV-I) from patients with AIDS and pre-AIDS. *Science* 1984;224:497–500.
3. Gallo RC, Salahuddin SZ, Popovic M, et al. Frequent detection and isolation of cytopathic retroviruses (HTLV-I) from patients with AIDS and at risk for AIDS. *Science* 1984;224:500–503.
4. Curran JW, Jaffe HW, Hardy AM, et al. Epidemiology of HIV infection and AIDS in the United States. *Science* 1988;239:610–616.
5. Schochetman G, George JR, editors. *AIDS testing: a comprehensive guide to technical, medical, social, legal, andmanagement issues*. 2nd ed. New York: NY Springer-Verlag; 1994.
6. Nduati R, John G, Mbori-Ngacha D, et al. Effect of breastfeeding and formula feeding on transmission of HIV-1: arandomized clinical trial. *Journal of the American Medical Association* 2000;283:1167–1174.
7. Perelson AS, Neumann AU, Markowitz M, Leonard JM, Ho DD. HIV-1 dynamics *in vivo*: virion clearance rate, infected cell life-span, and viral generation time. *Science* 1996; 271:1582–1586.
8. Wei X, Ghosh SK, Taylor ME, Johnson VA, Emini EA, Deutsch P, Lifson JD, Bonhoeffer S, Nowak MA, Hahn BH, et al. Viral dynamics in human immunodeficiency virus type 1 infection. *Nature* 1995; 373:117–122.
9. Ho DD, Neumann AU, Perelson AS, Chen W, Leonard JM, Markowitz M. Rapid turnover of plasma virions and CD4 lymphocytes in HIV-1 infection. *Nature* 1995; 373:123–126.
10. Katzenstein DA, Hammer SM, Hughes MD, Gundacker H, Jackson JB, Fiscus S, Rasheed S, Elbeik T, Reichman R, Japour A, Merigan TC, Hirsch MS. The relation of virologic and immunologic markers to clinical outcomes after nucleoside therapy in HIV-infected adults with 200 to 500 CD4 cells per cubic millimeter. AIDS Clinical Trials Group Study 175 Virology Study Team. *N Engl J Med* 1996; 335:1091–1098.
11. Mellors JW, Munoz A, Giorgi JV, Margolick JB, Tassoni CJ, Gupta P, Kingsley LA, Todd JA, Saah AJ, Detels R, Phair JP, Rinaldo CR, Jr. Plasma viral load and CD4+ lymphocytes as prognostic markers of HIV-1 infection. *Ann Intern Med* 1997; 126:946–954.
12. Mellors JW, Rinaldo CR, Jr., Gupta P, White RM, Todd JA, Kingsley LA. Prognosis in HIV-1 infection predicted by the quantity of virus in plasma. *Science* 1996; 272:1167–1170.
13. O'Brien WA, Hartigan PM, Martin D, Esinhart J, Hill A, Benoit S, Rubin M, Simberkoff MS, Hamilton JD. Changes in plasma HIV-1 RNA and CD4+ lymphocyte counts and the risk of progression to AIDS. Veterans Affairs Cooperative Study Group on AIDS. *N Engl J Med* 1996; 334:426–431.
14. Ruiz L, Romeu J, Clotet B, Balague M, Cabrera C, Sirera G, Ibanez A, Martinez-Picado J, Raventos A, Tural C, Segura A, Foz M. Quantitative HIV-1 RNA as a marker of clinical stability and survival in a cohort of 302 patients with a mean CD4 cell count of 300 x 10(6)/l. *Aids* 1996; 10:F39–44.
15. Saag MS, Holodniy M, Kuritzkes DR, O'Brien WA, Coombs R, Poscher ME, Jacobsen DM, Shaw GM, Richman DD, Volberding PA. HIV viral load markers in clinical practice. *Nat Med* 1996; 2:625–629.

VALIDATION

16. Centers for Disease Control and Prevention. *Biosafety in Microbiological and Biomedical Laboratories*. Richmond JY and McKinney RW (eds) (1993). HHS Publication number (CDC) 93-8395.
17. Clinical and Laboratory Standards Institute. *Protection of Laboratory Workers from Occupationally Acquired Infections*; Approved Guideline. Document M29 (refer to latest edition).
18. Clinical and Laboratory Standards Institute. *Evaluation of Detection Capability for Clinical Laboratory Measurement Procedures*; Approved Guideline. Document EP17-A2 (Second Edition). Wayne, PA: Clinical Laboratory Standards Institute; 2012.
19. Clinical and Laboratory Standards Institute. *Evaluation of the Linearity of Quantitative Measurement Procedures: AStatistical Approach*; Approved Guideline. NCCLS document EP06-A [ISBN 1-56238-498-8]. NCCLS, 940 West Valley Road, Suite 1400, Wayne, PA 19087-1898 USA, 2003.
20. Clinical and Laboratory Standards Institute. *Evaluation of Precision Performance of Clinical Chemistry Devices*; Approved Guideline. Document EP5-A2.

VALIDATION
