# Supplementary material for: Multidisease testing for HIV and TB using the GeneXpert platform: A feasibility study in rural Zimbabwe
Source: PLoS One. 2018 Mar 2;13(3):e0193577. doi: 10.1371/journal.pone.0193577 (PMC5834185; doi:10.1371/journal.pone.0193577)
Supplement: S2 Text — (PDF) [file pone.0193577.s005.pdf]

Standard Operating Procedures for HIV-1 Qualitative Early Infant Diagnostic testing using GeneXpert technology

July 2015

VALIDATION

Table of Contents

I. Title of Procedure 3
II. Test Summary 3
III. Principle 3
IV. Specimen Handling and Preparation 4
V. Quality Control 5
VI. Reagents, Materials, & Equipment 6
VII. Procedure 7
VIII. Interpretation of Results 9
IX. Possible reasons for repeating the assay 12
XII. References 13

Figure 1: Overview of the GeneXpert test cartridge 4
Figure 2: GeneXpert quality control result scenarios 5
Figure 3: GeneXpert transfer pipette and aerial view of the reaction cartridge 8
Figure 5: GeneXpert HIV-1 Qual Detected HIV 11
Figure 6: GeneXpert Qual HIV not detected 12

Table 1: GeneXpert HIV-1 Qual assay result interpretation 10

VALIDATION

I. Title of Procedure

Qualitative HIV-1 Early Infant Diagnostic testing using GeneXpert technology

II. Test Summary

Human Immunodeficiency Virus (HIV) is the etiologic agent of Acquired Immunodeficiency Syndrome (AIDS). It can be transmitted through sexual contact, exposure to infected blood or blood products, prenatal infection of a fetus, or perinatal or postnatal infection of a newborn.
Acute HIV infections typically last less than 14 days and are associated with high levels of viremia prior to a detectable immune response. Therefore, HIV-1 nucleic acid testing can be more sensitive than standard serologic testing in detection of acute infection. Early diagnosis of HIV infection in infants is a necessity; however, many patients are lost to follow-up while waiting for an Enzyme-Linked ImmunoSorbent Assay (ELISA), which is only accurate beginning in the 15 to 18 month age range. As a result, HIV-1 nucleic acid testing has been recommended for detecting infection in pediatric patients 18 months of age or younger.

The HIV-1 Qual Assay, performed on GeneXpert® Instrument Systems, is a qualitative nucleic acid amplification test designed to detect HIV-1 using human whole blood and dried blood spots (DBS). The HIV-1 Qual Assay uses reverse transcription polymerase chain reaction (RT-PCR) technology to achieve high sensitivity for the qualitative detection of HIV-1 total nucleic acids in whole blood or dried blood spot (DBS) specimen types. HIV diagnostics have evolved significantly in the past two decades and continue to be significantly important in saving lives of millions of HIV infected patients.

III. Principle

GeneXpert Instrument Systems automate and integrate sample preparation, nucleic acid extraction and amplification, and detection of the target sequence in simple or complex samples using real-time reverse transcriptase PCR (RT-PCR). The systems consist of an instrument, personal computer, and preloaded software for running tests and viewing the results. The systems require the use of single-use disposable GeneXpert cartridges that hold the RT-PCR reagents and host the RT-PCR processes. Because the cartridges are self-contained, cross-contamination between samples is minimized. The HIV-1 Qual Assay includes reagents for the detection of HIV-1 total nucleic acids in specimens as well as an internal control to ensure adequate processing of the target and to monitor the presence of inhibitors(s) in the RT and PCR reactions. The Probe Check Control (PCC) verifies reagent rehydration, PCR tube filling in the cartridge, probe integrity, and dye stability.

VALIDATION

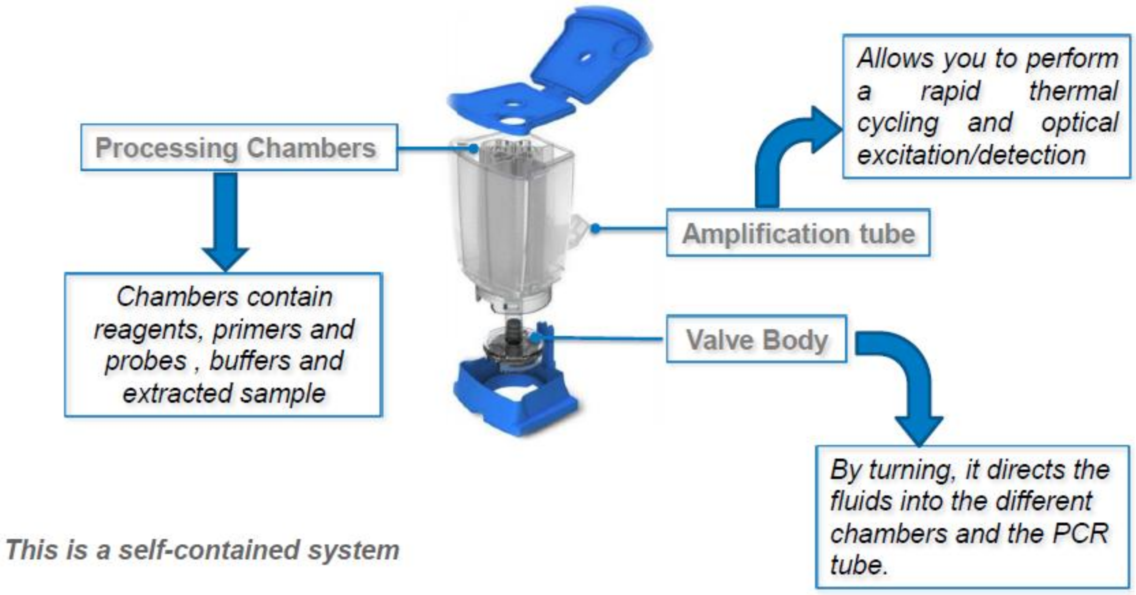

Figure 1: Overview of the GeneXpert test cartridge

IV. Specimen Handling and Preparation

- **<u>Whole blood</u>** should be collected in EDTA tubes and a minimum of 100ul of whole blood is required for the HIV-1 Qual Assay.
- EDTA anticoagulated blood may be stored at 20-35deg for up-to 8hours or at 2-8deg for up-to 72hours prior to preparing and testing the specimen.
- Dried Blood Spots (DBS) collect dried blood spot (DBS) specimens using appropriate clinical procedures. DBS should be prepared using Whatman 903filter paper cards or equivalent from blood obtained from a heel-, finger- or toe-stick or collected in an EDTA-tube. DBS are made by spotting blood inside each delineated 12-millimeter circle of the filter paper card. Ensure that the entire circle is covered with blood (approximately 60–70µL). A minimum of two circles should be made from each specimen to allow for retesting. If whole blood was collected in an EDTA-tube, mix the specimen by inverting it 8–10 times before applying it onto the filter. Air-dry the card at room temperature for a minimum of three hours. Package each card in individual resealable bags with a desiccant sachet in each bag. Freshly drawn specimens in EDTA-tubes may be held at 20–35 °C for up to 8 hours or at 2–8 °C for up to 72 hours, prior to making the DBS. Ship filter paper cards containing DBS to the testing laboratories for further processing in individual resealable bags with a desiccant sachet in each bag. The cards may be stored at 23–27 °C for up to four weeks. Alternatively, cards may be stored at 2–8 °C or -18 °C or colder for up to 12 weeks.

V. Quality Control

Each test includes a Sample Volume Adequacy (SVA), a Sample Processing Control (SPC) and Probe Check Control (PCC).

- **Sample Volume Adequacy (SVA):** Ensures that the sample was correctly added to the cartridge. The SVA verifies that the correct in-volume of sample has been added in the sample chamber. The SVA passes if it meets the validated acceptance criteria.
- **Sample Processing Control (SPC):** Ensures the sample was correctly processed. The SPC is an Armored RNA in the form of a dry bead that is included in each cartridge to verify adequate processing of the sample virus. The SPC verifies that lysis of HIV-1 has occurred if the organism is present and verifies that the specimen processing is adequate. Additionally this control detects specimen-associated inhibition of the RT-PCR reaction. The SPC should be positive in a negative sample and can be negative or positive in a positive sample. The SPC passes if it meets the validated acceptance criteria.
- **Probe Check Control (PCC):** Before the start of the PCR reaction, the GeneXpert Instrument System measures the fluorescence signal from the probes to monitor bead rehydration, reaction tube filling, probe integrity, and dye stability. The PCC passes if it meets the validated acceptance criteria.
- **External Controls:** External controls should be used in accordance with NMRL procedures and institutions with which they have an understanding (ZINQAP, CDC among other).

Xpert Assay and System Control
Summary

Procedural Step & Condition Controlled		IQS	Probe Check	System Checks	Final Results
Pre-, and during run	Heating, cooling, pressure (clogging), electronics, optics			X	Failure results in: ERROR
Sample Addition	Sample aspiration			X	Failure results in: ERROR
Sample Processing	Cell lysis	X			Failure results in: ERROR
	Nucleic acid recovery	X			Failure results in: ERROR
Reagent Addition	Bead Presence/Rehydration	X	X		Failure results in: ERROR or INVALID
	PCR tube fill	X	X		Failure results in: ERROR
	Fluorogenic detection	X	X		Failure results in: ERROR
PCR	Thermal cycling	X		X	Failure results in: ERROR
	Primer or target annealing	X			Failure results in: INVALID
	Inhibition	X			Failure results in: INVALID
Quantitation	Results Reported	X			Quantitative Value (IU/ml or copies /ml)<br>Detected Above range<br>Detected Below Range<br>Not Detected

Figure 2: GeneXpert quality control result scenarios

VI. Reagents, Materials, & Equipment

The HIV-1 Qual Assay kit contains sufficient reagents to process 10 specimens or quality control samples. The kit contains the following:

1. 10 HIV-1 Qual Assay Cartridges with Integrated Reaction Tubes. Each cartridge contains:
   - Bead 1, Bead 2, and Bead 3 (freeze-dried)
   - Lysis Reagent (Guanidinium Thiocyanate) 2.0 mL
   - Rinse Reagent 0.5 mL
   - Elution Reagent 1.5 mL
   - Binding Reagent 2.4 mL
   - Proteinase K Reagent 0.48 mL
2. 10 HIV-1 Qual sample reagent set (sample reagent)
3. Disposable 1 mL Transfer Pipettes 10 per kit
4. Disposable 100ul transfer pipettes
5. CD 1 per kit
   - Assay Definition File (ADF)
   - Instructions to import ADF into GeneXpert software
   - Package Insert

Storage and Handling

- Store the HIV-1 Qual Assay cartridges at 2–28 °C.
- Do not open the cartridge lid until you are ready to perform the test.
- Use cartridge within four hours after opening the cartridge lid.
- Do not use a cartridge that has leaked or become cloudy.

Materials Required but Not Provided

- Printer
- Bleach
- Eppendorf themomixer and smartblock
- Forceps, bleach and serviettes
- DBS collection kit, sterile scissors

Warnings and Precautions

- Treat all biological specimens, including used cartridges, as if capable of transmitting infectious agents. Because it is often impossible to know which might be infectious, all biological specimens should be treated with standard precautions.
- Do not open the HIV-1 Qual Assay cartridge lid except when adding the Sample reagent and whole blood or the Sample reagent DBS sample.
- Do not use a cartridge if it appears wet or if the seal appears to have broken nor a cartridge that has been dropped after removing it from packaging
- Do not shake the cartridge. Shaking or dropping the cartridge after opening the lid may yield invalid results.
- Do not place the sample ID label on the cartridge lid or on the barcode label.
- Each single-use HIV-1 Qual Assay cartridge is used to process one specimen. Do not reuse spent cartridges.

VALIDATION

- Do not use a cartridge that has a damaged reaction tube.
- Single-use disposable pipette is used to transfer one specimen. Do not reuse disposable pipettes.
- Wear clean lab coats and gloves. Change gloves between processing each sample.
- In the event of contamination of the work area or equipment with samples or controls, thoroughly clean the contaminated area with a solution of 1:10 dilution of household chlorine bleach and then 70% ethanol. Wipe work surfaces dry completely before proceeding.

VII. Procedure

Before starting, remove the vial containing the sample reagent from the kit and allow to adjust to room temperature. See Figure 1 below. If the vial has not been stored in an upright position, make sure the buffer is settled in the bottom by giving the vial a firm shake.

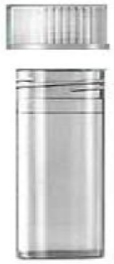

Figure 1: HIV-1 Qual assay vial Sample reagent

1. Preparing the Cartridge

<u>Whole blood</u>

- Wear protective disposable gloves.
- Inspect the test cartridge for damage. If damaged, do not use it.

Note *There is a thin plastic film that covers the inner ring of 13 ports of the test cartridge. This film should not be removed*

- Label the test cartridge with the patient sample ID number
- Open the lid of the test cartridge.
- Use the 1 mL transfer pipette provided or an automatic pipette to transfer 750µL of the sample reagent into the sample chamber of the cartridge (Figure 3).

• **Option 1:** If using the transfer pipette included in the kit (Figure 1), fill to just below the bulb but above the line to transfer at least 1 mL plasma from the collection tube into the sample chamber of the test cartridge (Figure 2). Do **NOT** pour the specimen into the chamber!

VALIDATION

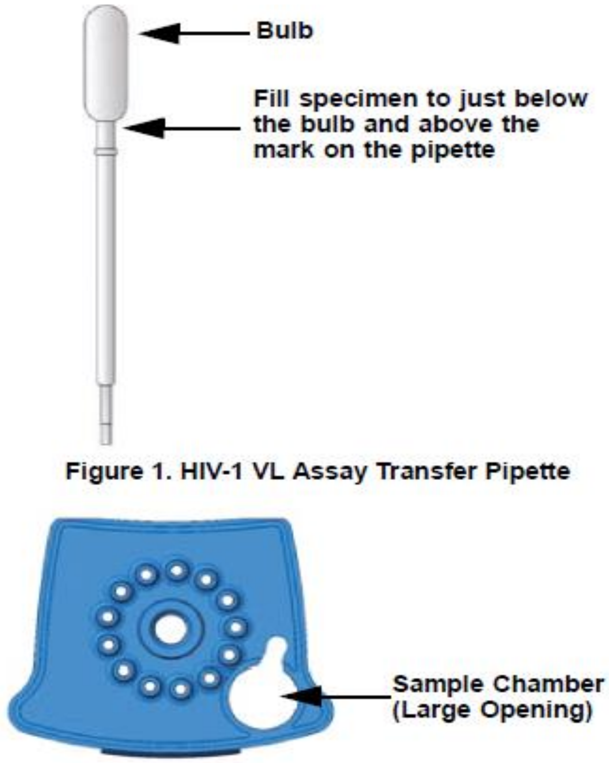

Figure 3: GeneXpert transfer pipette and aerial view of the reaction cartridge

- Close the cartridge lid.
- Load the cartridge into the GeneXpert instrument or Infinity system.

Dried Blood Spots

1. Wear protective disposable gloves.
2. Turn on Thermomixer to heat to 56 °C.
3. Label the Sample Reagent vial with the specimen identification.
4. Using sterilized scissors, excise one entire DBS from the filter paper card for each specimen. Follow the delineated lines when excising the DBS. If perforated circles are used, use forceps to detach the DBS.
5. Unscrew the lid on the vial containing the Sample Reagent and place one DBS in the vial. If DBS does not settle to the bottom, use the backside of the forceps to gently push it down. Ensure that the DBS is fully submerged in the Sample Reagent buffer.
6. Place the vial with the DBS in a Thermomixer and incubate for 15 minutes, at 56 °C and rotate at 500 rpm.
7. Inspect the test cartridge for damage. If damaged, do not use.
8. Open the cartridge lid.
9. Use the 1 mL transfer pipette provided (above) or an automatic pipette to transfer all the liquid from the lysed DBS specimen into the sample chamber of the cartridge (Figure 3). Ensure the pipette is filled above the third mark on the transfer pipette. Avoid suction of the DBS with the pipette. Do **NOT** pour the specimen into the chamber!
10. Close the cartridge lid.

Important Start the test within four hours of adding the sample to the cartridge

- ✓ **Important Before starting the test, make sure the HIV-1 Qual Assay Definition File (ADF) is imported into the software**

Starting the Test

This section lists the basic steps for running the test. For detailed instructions, see the GeneXpert System Operator Manual on the computer desktop.

a. Turn on the GeneXpert instrument. First turn on the instrument and then turn on the computer. The GeneXpert software will launch automatically. If it doesn't, double-click the GeneXpert

b. Software shortcut icon on the Windows® desktop.

c. Log on to the GeneXpert Instrument System software using your user name and password. Click Create Test

d. Scan in the Patient ID (optional). If typing the Patient ID, make sure the Patient ID is typed correctly. The Patient ID is associated with the test results and is shown in the View Results window.

e. Scan or type in the Sample ID. If typing the Sample ID, make sure the Sample ID is typed correctly. The Sample ID is associated with the test results and is shown in the View Results window and all reports. The Scan Cartridge dialog box appears.

f. Scan the barcode on the Xpert® HIV-1 Qual assay cartridge. The Create Test window appears. Using the barcode information, the software automatically fills the boxes for the following fields: Select Assay, Reagent Lot ID, Cartridge SN, and Expiration Date.

g. Click Start Test. Enter your password, if requested.

h. Open the instrument module door with the blinking green light and load the cartridge.
   - Close the door. The test starts and the green light stops blinking. When the test is finished, the light turns off.
   - Wait until the system releases the door lock before opening the module door and removing the cartridge.
   - The used cartridges should be disposed in the appropriate specimen waste containers according to your institution's standard practices.

- ➢ **WARNING: GeneXpert HIV Qual and Quant cartridges contain a highly toxic chemical (to human and environment), Guanidinium Thiocyanate, and should be disposed of after use, in an incinerator capable of reaching 1200°C.**

VIII. Interpretation of Results

Click the View Results icon to view results.

Upon completion of the test, click the **Report** button of the **View Results** window to view and/or generate a PDF report file. The test results take approximately 90minutes to be produced

VALIDATION

The results are interpreted automatically by the GeneXpert Instrument System from measured fluorescent signals and embedded calculation algorithms and are clearly shown in the View Results window (Figure 5 and Figure 6). Possible results are shown in Table 1.

Table 1: GeneXpert HIV-1 Qual assay result interpretation

Result	Interpretation
**HIV-1 DETECTED**<br>See Figure 5.	The HIV-1 target nucleic acids are detected.<br>• The HIV-1 target nucleic acids have a Ct within the valid range and endpoint above the minimum setting.<br>• SPC: NA (not applicable); SPC is ignored because the HIV-1 target amplification occurred.<br>• Probe Check: PASS; all probe check results pass.
**HIV-1 NOT DETECTED**<br>See Figure 6.	The HIV-1 target nucleic acids are not detected. SPC meets acceptance criteria.<br>• SPC: PASS; SPC has a Ct within the valid range and endpoint above the minimum setting.<br>• Probe Check: PASS; all probe check results pass.
**INVALID**	Presence or absence of the target nucleic acids cannot be determined. Repeat test according to the instructions in Section 15.2, Retest Procedure.<br>• SPC: FAIL; SPC Ct is not within valid range and the endpoint is below the minimum setting.<br>• Probe Check: PASS; all probe check results pass.
**ERROR**	Presence or absence of HIV-1 target nucleic acids cannot be determined. Repeat test according to the instructions in Section 15.2, Retest Procedure<br>• HIV-1: NO RESULT<br>• SPC: NO RESULT<br>• Probe Check: FAIL*; all or one of the probe check results fail.<br>* If the probe check passes, the error is caused by the maximum pressure limit exceeding the acceptable range or by a system component failure.
**NO RESULT**	Presence or absence of HIV-1 target nucleic acids cannot be determined. Repeat test according to the instructions in Section 15.2, Retest Procedure. A **NO RESULT** indicates that insufficient data were collected. For example, the operator stopped a test that was in progress.<br>• HIV-1: NO RESULT<br>• SPC: NO RESULT<br>• Probe Check: NA (not applicable).

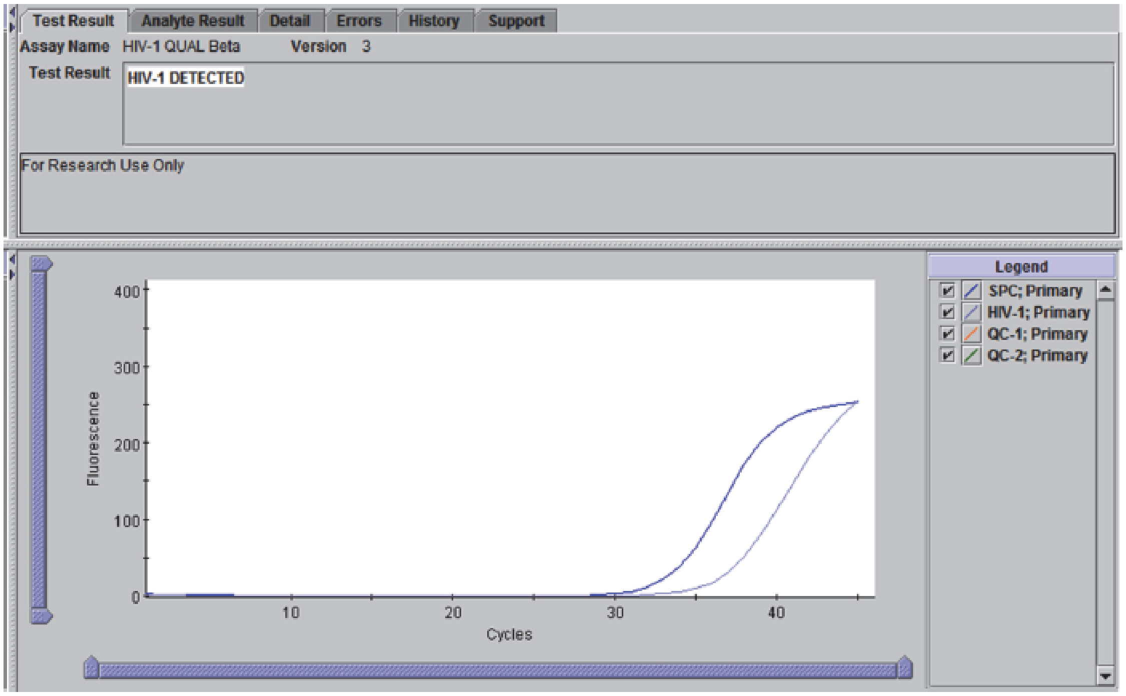

Figure 4: GeneXpert HIV-1 Qual Detected HIV

VALID

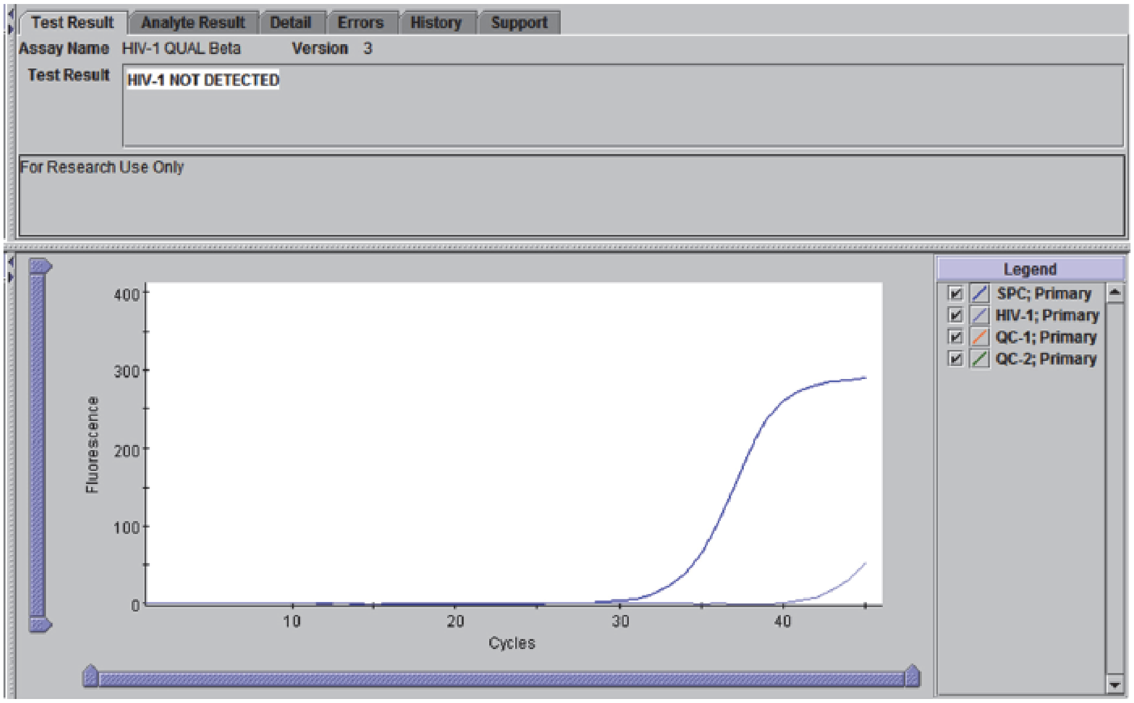

Figure 5: GeneXpert Qual HIV not detected

IX. Possible reasons for repeating the assay

•An INVALID result indicates that the control SPC failed or the sample was not properly processed or PCR was inhibited.

•An ERROR result indicates that the assay was aborted. Possible causes include: insufficient volume of sample was added, a reagent probe integrity problem was detected, or the maximum pressure limit was exceeded.

•A NO RESULT indicates that insufficient data were collected. For example, the operator stopped a test that was in progress, a load error occurred, or the software was closed prematurely.

Retest Procedure

For retest of an **INVALID**, **ERROR**, or **NO RESULT**, use a new cartridge and new reagents (do not re-use the cartridge).

1. Remove a new cartridge from the kit.
2. Section VII on preparing cartridge and starting the test on page 7.

TECHNICAL SUPPORT
Raynolds Mangena
Pointe care diagnostics
Harare
+263 77 290 7343

Chepeid
South Africa
+27 11 234 9636
MOBILE +27 71 209 8069
FAX +27 11 234 9640
EMAIL dipti.lallubhai@cepheid.com

VALIDA

XII. References

1. Barre-Sinoussi F, Chermann JC, Rey F, et al. Isolation of a T-lymphotropic retrovirus from a patient at risk for acquired immune deficiency syndrome (AIDS). Science 1983;220:868–871.
2. Popovic M, Sarngadharan MG, Read E, et al. Detection, isolation and continuous production of cytopathic retroviruses (HTLV-I) from patients with AIDS and pre-AIDS. Science 1984;224:497–500.
3. Gallo RC, Salahuddin SZ, Popovic M, et al. Frequent detection and isolation of cytopathic retroviruses (HTLV-I) from patients with AIDS and at risk for AIDS. Science 1984;224:500–503.
4. Curran JW, Jaffe HW, Hardy AM, et al. Epidemiology of HIV infection and AIDS in the United States. Science 1988;239:610–616.
5. Schochetman G, George JR, editors. AIDS testing: a comprehensive guide to technical, medical, social, legal, and management issues. 2nd ed. New York: NY Springer-Verlag; 1994.
6. Nduati R, John G, Mbori-Ngacha D, et al. Effect of breastfeeding and formula feeding on transmission of HIV-1: a randomized clinical trial. Journal of the American Medical Association 2000;283:1167–1174.
7. Kahn JO, Walker BD. Acute human immunodeficiency virus type 1 infection. New England Journal of Medicine 1998;339:33–39.
8. Gaines H, von Sydow M.A, von Stedingk LV. Immunological changes in primary HIV-1 infection. AIDS 1990;4:995–999.

9. Tindall B, Cooper DA. Primary HIV-1 infection: host responses and intervention strategies. AIDS 1991;5:1–14.
10. Daar ES, Moudgil T, Meyer RD, Ho DD. Transient high levels of viremia in patients with primary human immunodeficiency virus type 1 infection. New England Journal of Medicine 1991;324:961–964.
11. Clark SJ, Saag MS, Decker WD. High titers of cytopathic virus in plasma of patients with symptomatic primary HIV-1 infection. New England Journal of Medicine 1991;324:954–960.
12. Joint United Nations Programme on HIV/AIDS (UNAIDS). 2012 Report on the global AIDS epidemic. http://www.unaids.org/en/media/unaids/contentassets/documents/epidemiology/2012/gr2012/20121120_UNAIDS_Global_Report_2012_en.pdf. Published 2012. Accessed January 4, 2013.
13. Hankins C. Overview of the Current State of the Epidemic. Current HIV/AIDS Reports 2013;10(2):113–123.
14. Brahmbhatt H, Kigozi G, Wabire-Mangen F, et al. Mortality in HIV-infected and uninfected children of HIV-infected and uninfected mothers in rural Uganda. Journal of Acquired Immune Deficiency Syndromes 2006;41(4):504–508.
15. Marinda E, Humphrey JH, Iliff PJ, et al. Child mortality according to maternal and infant HIV status in Zimbabwe. Pediatric Infectious Disease Journal 2007;26(6):519–526.
16. Newell ML, Coovadia H, Cortina-Borja M, et al. Mortality of infected and uninfected infants born to HIV-infected mothers in Africa: a pooled analysis. Lancet 2004;364(9441):1236–1243.
17. Shetty AK. Epidemiology of HIV Infection in Women and Children: A Global Perspective. Current HIV Research 2013;11(2):81–92.
18. Sherman GG, Cooper PA, Coovadia AH, et al. Polymerase chain reaction for diagnosis of human immunodeficiency virus infection in infancy in low resource settings. Pediatric Infectious Disease Journal 2005;24(11):993–997.
19. Sherman GG, Matsebula TC, Jones SA. Is early HIV testing of infants in poorly resourced prevention of mother to child transmission programmes unaffordable? Tropical Medicine & International Health 2005;10(11):1108–1113.
20. Read JS. Committee on Pediatric AIDS, American Academy of Pediatrics. Diagnosis of HIV-1 infection in children younger than 18 months in the United States. Pediatrics 2007;120:e1547–1562.
21. Prendergast A, Tudor-Williams G, Jeena P, et al. International perspectives, progress, and future challenges of paediatric HIV infection. Lancet 2007;370:68–80.
22. World Health Organization. Antiretroviral therapy for HIV infection in infants and children: towards universal access, recommendations for a public health approach. Geneva: World Health Organization; 2006.
23. Global AIDS Alliance. Scaling up access to early infant diagnostics: accelerating progress through public-private partnerships. Washington DC: Global AIDS Alliance; 2008.
24. Centers for Disease Control and Prevention. Biosafety in Microbiological and Biomedical laboratories. Richmond JY and McKinney RW (eds) (1993). HHS Publication number (CDC) 93-8395.
25. Clinical and Laboratory Standards Institute (formerly National Committee for Clinical Laboratory Standards). Protection of Laboratory Workers from Occupationally Acquired Infections; Approved Guideline. Document M29 (refer to latest edition).

VALIDATION
