# Supplementary material for: Multidisease testing for HIV and TB using the GeneXpert platform: A feasibility study in rural Zimbabwe
Source: PLoS One. 2018 Mar 2;13(3):e0193577. doi: 10.1371/journal.pone.0193577 (PMC5834185; doi:10.1371/journal.pone.0193577)
Supplement: S3 Text — (PDF) [file pone.0193577.s006.pdf]

Standard Operating Procedures for MTB/Rif testing using GeneXpert technology

Sept 2013

VALIDATION

Table of Contents

I. Title of Procedure 3
2. Principle 3
IV. Specimen Handling and Preparation 3
V. Quality Control .... **Error! Bookmark not defined.**
VI. Reagents, Materials, & Equipment 3
VII. Procedure 4

VALIDATION

I. Title of Procedure

Rapid testing for Mycobacterium tuberculosis and determining Rifampicin resistance from sputum samples using GeneXpert technology

2. Principle

This diagnostic test (also known as the Xpert M.tb/RIF) test is based on a semi-quantitative, nested real-time PCR for the detection of M. tuberculosis complex DNA in sputum samples and the detection of rifampicin (RIF) resistance associated mutations of the rpoB gene in samples from patients at risk for RIF resistance. The M.tb/RIF test is intended for use with specimens from untreated patients for whom there is clinical suspicion of tuberculosis, so that the results can be provided within 2 hours. Basic step by step instructions are detailed below required to perform the test. For a full description of the system, protocol (e.g. preparing the cartridge) and QC procedures as well as how to visualize figures and software settings see the Xpert M.tb/RIF System Operator Manual

3. Specimen Handling and Preparation

Each patient was asked to produce two sputum specimens and they were submitted to the laboratory immediately.
The laboratory assessed:

- sputum condition (i.e. the sample does not contain only saliva, or excessive blood quantity and is of appropriate volume),
- Specimen Transfer Form completion

The samples were within 48 hours of sample collection and where samples couldn't be processed on the same day, they were stored in the refrigerator (2-8 °C).

4. Reagents, Materials, & Equipment

- GeneXpert Dx System equipped with GX2.1 software (catalogue number varies by configuration):
-GeneXpert instrument, computer, barcode wand reader, and Operator Manual
- Lockable container with appropriate disinfectant
- Micro tube 1.5 mL
- Rack able to hold 15 mL Falcon tubes
- Sterile Falcon tubes (16.5x120 mm, 15 mL)
- Sterile transfer pipettes (3.5 mL)
- Timer
- Vortex Mixer
- Xpert M.tb/RIF kit (CGXM.tb/RIF-10) [contains sufficient reagents to process 10 patient or quality-control specimens]

VALIDATION

Storage and Handling

- Store the M.tb/RIF Assay cartridges at 2–8 °C.
- Do not open the cartridge lid until you are ready to perform the test.
- Use cartridge within four hours after opening the cartridge lid.
- Do not use a cartridge that has leaked.

Materials Required but Not Provided

- Printer
- Bleach

Warnings and Precautions

- Treat all biological specimens, including used cartridges, as if capable of transmitting infectious agents. Because it is often impossible to know which might be infectious, all biological specimens should be treated with standard precautions.
- Do not open the M.tb/RIF Assay cartridge lid until you are ready to add the specimen
- Do not use a cartridge that has been dropped after removing it from the packaging.
- Do not shake the cartridge. Shaking or dropping the cartridge after opening the lid may yield invalid results.
- Do not place the sample ID label on the cartridge lid or on the barcode label.
- Each single-use M.tb/RIF VL Assay cartridge is used to process one specimen. Do not reuse spent cartridges.
- Do not use a cartridge that has a damaged reaction tube.
- Single-use disposable pipette is used to transfer one specimen. Do not reuse disposable pipettes.
- Wear clean lab coats and gloves. Change gloves between processing each sample.
- In the event of contamination of the work area or equipment with samples or controls, thoroughly clean the contaminated area with a solution of 1:10 dilution of household chlorine bleach and then 70% ethanol. Wipe work surfaces dry completely before proceeding.

5. Procedure

Step One: Preparing the Sputum sample

1. Sputum processing has two major functions: sputum digestion (liquefaction) of organic debris in the specimen and decontamination of bacteria other than mycobacteria.
2. The GeneXpert sample reagent was added into the sputum container (2:1 v/v), vigorously mixed and incubated for 15 minutes at room temperature.

Step Two: Preparing the Cartridge

1. Label each Xpert *M.tb*/RIF cartridge with the lab accession number by writing on the sides of the cartridge or attach ID label. Note: do not put the label on the lid or obstruct the existing 2D barcode on the cartridge.

VALIDATION

2. Using the sterile transfer pipette provided with the kit, aspirate the liquefied sample into the transfer pipette until the meniscus is above the minimum mark. **Do not process the sample further if there is insufficient volume.**
3. Open the cartridge lid and transfer sample into the open port of the Xpert *M.tb*/RIF cartridge. Dispense sample slowly to minimize risk of aerosol formation.
4. Close the cartridge lid and make sure the lid snaps firmly into place. Remaining liquefied sample may be kept for up to 12 hours at 2 – 8 ºC should repeat testing be required.
5. Be sure to load the cartridge into the GeneXpert Dx instrument and start the test within 30 minutes of preparing the cartridge.

Step Three: Starting the Test

1. Before you start the test, ensure the system is equipped with the GX2.1 software AND the Xpert *M.tb*/RIF assay is imported into the software.
2. Turn on the computer, followed by the GeneXpert Dx instrument (if not already on).
3. On the Windows™ desktop, double-click the GeneXpert Dx shortcut icon.
4. Log on to the GeneXpert Dx System software using your user name and password.
5. In the GeneXpert Dx System window, click **Create Test**. The Scan Cartridge Barcode dialog box appears.
6. Scan the 2D barcode located on the Xpert *M.tb*/RIF cartridge. The Create Test window appears. The software will automatically fill the boxes for the following fields: Select Assay, Reagent Lot ID, Cartridge SN, and Expiration Date based on the barcode information.
7. In the **Sample ID** box, scan or type the sample lab accession number. Cross-check to ensure it is typed or scanned correctly. The sample ID/lab accession number is associated with the test results in the "**View Results**" window and all generated reports.
8. Click **Start Test**. In the dialog box that appears, type your password.
9. Open the instrument module door with the flashing green light and load the cartridge.
10. Close the door. The green light will stop flashing and become steady once the test starts. When the test is finished, the green light will turn off and the system will release the door lock.
11. Once the system releases the door lock at the end of the run, open the module door and remove the cartridge.
12. Used cartridges are considered capable of transmitting infectious agents. Dispose the used cartridges according to your institution's and country's safety guidelines.

6. Quality Control

Each test includes a Sample Processing Control (SPC) and probe check (PCC):
- Sample Processing Control (SPC):
Ensures the sample was correctly processed. The SPC contains non-infectious spores in the form of a dry spore cake that is included in each cartridge to verify adequate processing of *M.tb*. The SPC verifies that lysis of *M.tb* has occurred if the organisms are present and verifies that specimen processing is adequate.
Additionally, this control detects specimen associated inhibition of the real-time PCR assay. The SPC should be positive in a negative sample and can be negative or positive in a positive sample.

VALIDATION

The SPC passes if it meets the validated acceptance criteria. The test result will be “Invalid” if the SPC is not detected in a negative test.

Probe Check Control (PCC):
Before the start of the PCR reaction, the GeneXpert Dx System measures the fluorescence signal from the probes to monitor bead rehydration, reaction-tube filling, probe integrity and dye stability. Probe Check passes if it meets the assigned acceptance criteria.

7. Interpretation, Recording and Reporting of Results

The results are interpreted by the GeneXpert DX System from measured fluorescent signals and embedded calculation algorithms and will be displayed in the “**View Results**” window of the GeneXpert machine.

M.tb Detected
M.tb target DNA is detected.
- *M.tb* Detected - The *M.tb* result will be displayed as High, Medium, Low or Very Low depending on the Ct value of the *M.tb* target present in the sample.
- Rif Resistance DETECTED, Rif Resistance NOT DETECTED, or Rif Resistance INDETERMINATE will be displayed only in *M.tb* DETECTED results and will be on a separate line from the *M.tb* DETECTED result.
- Rif Resistance DETECTED; a mutation in the *rpoB* gene has been detected that falls within the valid delta Ct setting.
- Rif Resistance INDETERMINATE; the *M.tb* concentration was very low and resistance could not be determined.
- Rif Resistance NOT DETECTED; no mutation in the *rpoB* gene has been detected.
- SPC– NA (not applicable); SPC signal is not required since *M.tb* amplification may compete with this control.
- Probe Check–PASS; all probe check results pass.

M.tb Not Detected
M.tb target DNA is not detected, SPC meets acceptance criteria.
- *M.tb* NOT DETECTED—*M.tb* target DNA is not detected.
- SPC– Pass; SPC has a Ct valid range and endpoint above the endpoint minimum setting.
- Probe Check–PASS; all probe check results pass.

RIF Not Detected
RIF target DNA is not detected, SPC meets acceptance criteria.
- RIF NOT DETECTED—RIF target DNA is not detected
- SPC– Pass; SPC has a Ct valid range and endpoint above the endpoint minimum setting.
- Probe Check–PASS; all probe check results pass.

VALIDATION

INVALID
Presence or absence of *M.tb* cannot be determined, repeat test with extra specimen. SPC does not meet acceptance criteria, the sample was not properly processed, or PCR is inhibited.
- *M.tb* INVALID–Presence or absence of *M.tb* DNA cannot be determined.
- SPC–FAIL; *M.tb* target result is negative and the SPC Ct is not within valid range.
- Probe Check–PASS; all probe check results pass.

ERROR
- *M.tb*–NO RESULT
- SPC–NO RESULT
- Probe Check–FAIL*; one or more of the probe check results fail.

*If the probe check passed, the error is caused by a system component failure.

NO RESULT
- *M.tb*–NO RESULT
- SPC–NO RESULT

8. Reasons to Repeat the Assay

Repeat the test using a new cartridge or initiate alternate procedures if one of the following test results occurs:
- An INVALID result indicates that the SPC failed. The sample was not properly processed or PCR was inhibited.
- An ERROR result indicates that the Probe Check control failed and the assay was aborted possibly due to the reaction tube being filled improperly, a reagent probe integrity problem was detected, or because the maximum pressure limits were exceeded or there was a GeneXpert module failure.
- A NO RESULT indicates that insufficient data were collected. For example, the operator stopped a test that was in progress.
- Rifampicin resistance is indeterminate.

VALIDATION

TECHNICAL SUPPORT

Raynolds Mangena
Pointe care diagnostics
Harare
+263 77 290 7343

Chepeid
South Africa
+27 11 234 9636

MOBILE +27 71 209 8069

FAX +27 11 234 9640

EMAIL dipti.lallubhai@cepheid.com

VALIDA
